# Antioxidant Activity of Compounds Isolated from *Elaeagnus umbellata* Promotes Human Gingival Fibroblast Well-Being

**DOI:** 10.1021/acs.jnatprod.9b01030

**Published:** 2020-02-07

**Authors:** Anna Maria Iannuzzi, Chiara Giacomelli, Marinella De Leo, Deborah Pietrobono, Fabiano Camangi, Nunziatina De Tommasi, Claudia Martini, Maria Letizia Trincavelli, Alessandra Braca

**Affiliations:** † Dipartimento di Farmacia, Università di Pisa, Via Bonanno 33, 56126 Pisa, Italy; ‡ Centro Interdipartimentale di Ricerca “Nutraceutica e Alimentazione per la Salute”, Università di Pisa, Via del Borghetto 80, 56124 Pisa, Italy; § Scuola Superiore Sant’Anna di Studi Universitari e di Perfezionamento, Piazza Martiri della Libertà 33, 56127 Pisa, Italy; ⊥ Dipartimento di Farmacia, Università degli Studi di Salerno, Via Giovanni Paolo II 132, 84084 Fisciano (SA), Italy

## Abstract

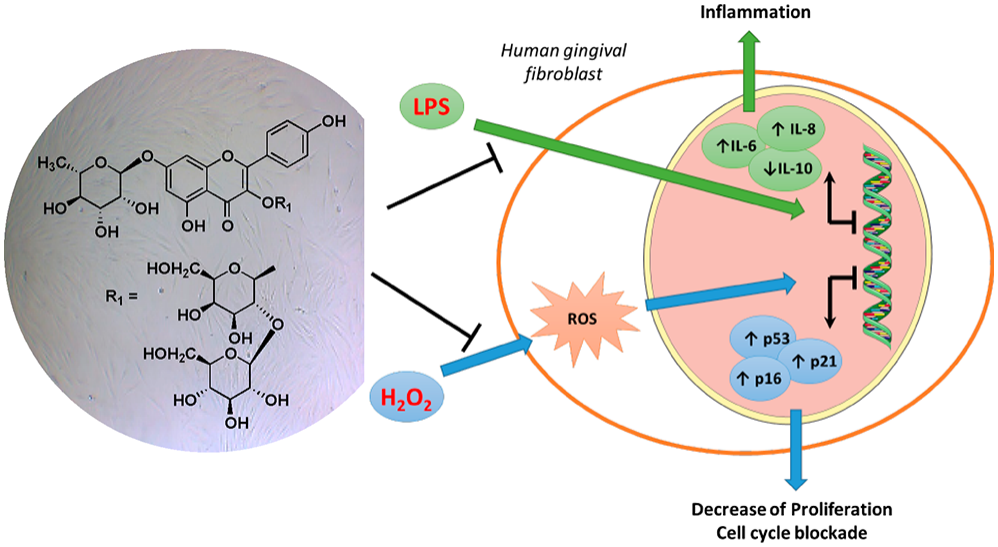

Four new triterpenoid bidesmosidic saponins (**1**–**4**) and a sesquiterpenoid glucoside (**5**), together with nine known phenolic compounds (**6**–**14**), were isolated from the fruits of *Elaeagnus umbellata*. Their structures were elucidated using 1D and 2D NMR spectroscopy and mass spectrometry data. The antioxidant capability of the isolated compounds was evaluated in human gingival fibroblasts. Compound **6** decreased ROS production and promoted cell proliferation. It also counteracted the cell cycle blockade induced by a low concentration of H_2_O_2_ decreasing the expression of p21 and CDKN2A (p16^INK4A^). Compound **6** decreased the expression of inflammatory cytokines (IL-6 and IL-8) in response to inflammatory stimuli, supporting its possible use in periodontitis lesions.

The genus *Elaeagnus* (Elaeagnaceae) includes about 90 species spread around the world, particularly in the temperate and subtropical regions of Asia, most of which are considered as folk medicinal plants for their pharmacological effects.^1^ Among these species, *Elaeagnus umbellata* Thunb, also known as “cardinal olive” or “autumn olive”, is a small deciduous shrub that grows as a tree 5–6 m tall, native to Southern Europe and Central Asia and then introduced to North America as an ornamental plant.^2^ In Indian traditional medicine, *E. umbellata* flowers are used in cardiac and respiratory diseases, while the seed oil is used for the treatment of pulmonary infections, coughs, and cardiac ailments.^3^ In Japan and China, the leaves are used as a tonic and their decoction is used to treat bowel disorders.^4^ The fruits, produced in great quantities and ripening between September and November, are small, round, juicy, reddish to pink, dotted with scales, and carrying a single seed.^5^ The fruits are edible, but not widely cultivated and consumed as a food, except for some areas of Asia, where the fruits are incorporated into the common diet, due to their benefits against hepatitis, fractures, injuries, and diarrhea, and used to prepare juices, jams, preserves, and other food stuffs.^6^ The red pigmentation of the berries is due to the presence of a large amount of carotenoids, especially lycopene (about 5–20 times higher than that of the ordinary tomato), which is reported to have anticancer, antioxidant, hepatoprotective, and cardioprotective effects.^5,6^ Therefore, these fruits have recently been used for the development of lycopene-rich extracts or powders for food formulation.^5^ In Italy, the plant is nonindigenous and is cultivated for ornamental purposes, while the fruits are eaten fresh, a custom that was imported from Asia.^7^ Few phytochemical investigations on *E. umbellata* berries are reported describing the presence of bioactive compounds such as polyphenols and flavonoids, which contribute in part to the in vitro antioxidant activity of “cardinal olive” extracts,^2^ and also alkaloids, steroids, terpenoids, and saponins.^8^

Thus, the present work was carried out to fully investigate the chemical profile of *E. umbellata* fruits cultivated in Italy, leading to the isolation and structural characterization of five new compounds, including four triterpenoid saponins (**1**–**4**) and one sesquiterpenoid glucoside (**5**), together with nine known phenolics (**6**–**14**). It is well known that flavonoid aglycones and corresponding glycosides possess antioxidant and anti-inflammatory activities.^9−11^ Oxidative stress and related inflammatory processes play crucial roles in different pathologies of the oral cavity such as periodontitis and oral mucositis. Periodontitis is a common inflammatory disease that derives from bacterial infection and proceeds with an abnormal host response. It can result in the destruction of oral soft tissues and can affect systemic health.^12^ Oral mucositis is one of the most common side effects observed during cancer radiotherapy and chemotherapy treatment.^13^ The modulation of reactive oxygen species (ROS) has gained attention due to their pivotal role in the progression of oral inflammatory diseases.^14^ ROS are a group of molecules derived by the physiological metabolism of oxygen in cells, and intracellular ROS such as hydrogen peroxide (H_2_O_2_) have been widely investigated for their controversial role. In the host response to bacterial infection, the activation of neutrophils promotes ROS production^15^ that causes the damage of different macromolecules, leading to a premature aging of periodontal tissue.^16^ Similarly, the production of ROS by radiotherapy and chemotherapy plays a pivotal role in the initiation phase of oral mucositis.^17^ Recently, several efforts have been made to discover synthetic molecules, natural extracts, and compounds that could locally counteract the production of ROS and the insurgence of inflammatory processes.^18−20^ Interestingly, the healing effects of an aqueous ethanolic extract of *E. angustifolia* has been reported in 5-fluorouracil-induced oral mucositis in male golden hamsters.^21^ Considering the possibility to consume the *E. umbellata* fruits, the antioxidant ability and the beneficial effects of their extracts and isolated compounds (**2** and **5**–**14**) in human gingival fibroblasts were evaluated for the first time. Furthermore, the ability of the most promising derivative, compound **6**, to decrease the transcription of lipopolysaccharide (LPS)-induced pro-inflammatory cytokines (IL-6 and IL-8) was also assessed.

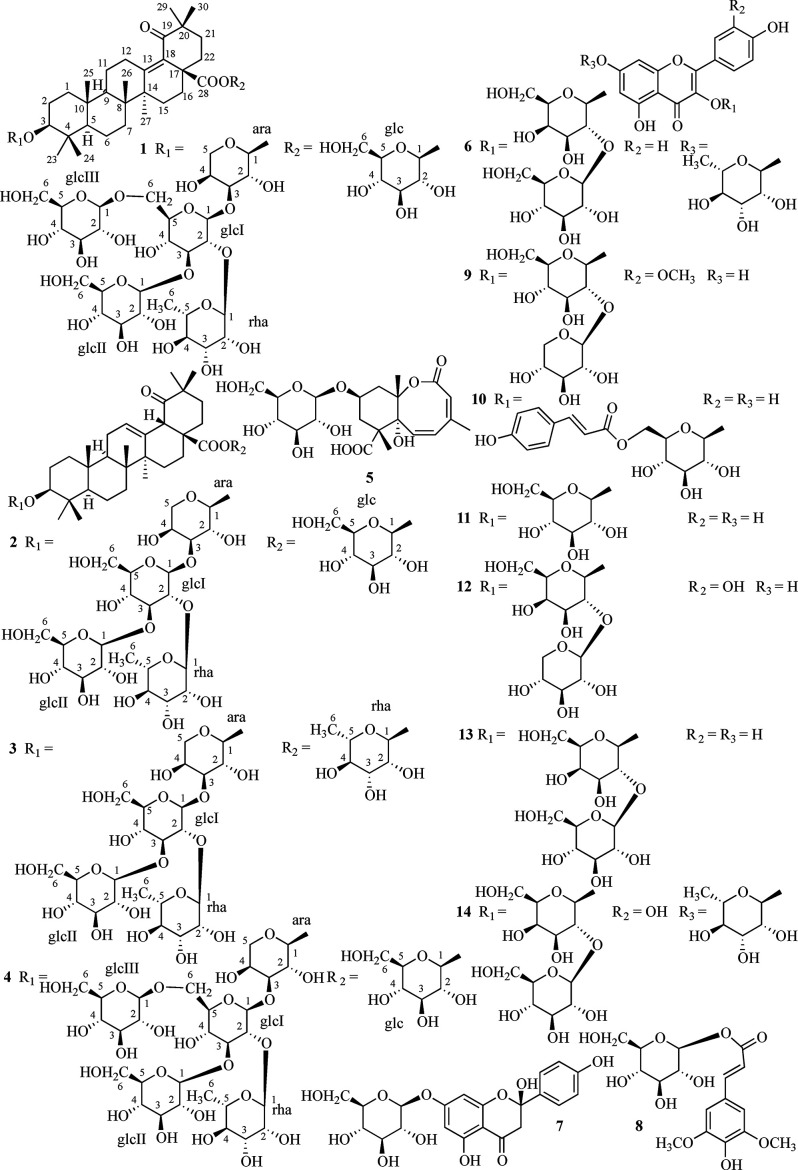



## Results and Discussion

**Figure 1:**
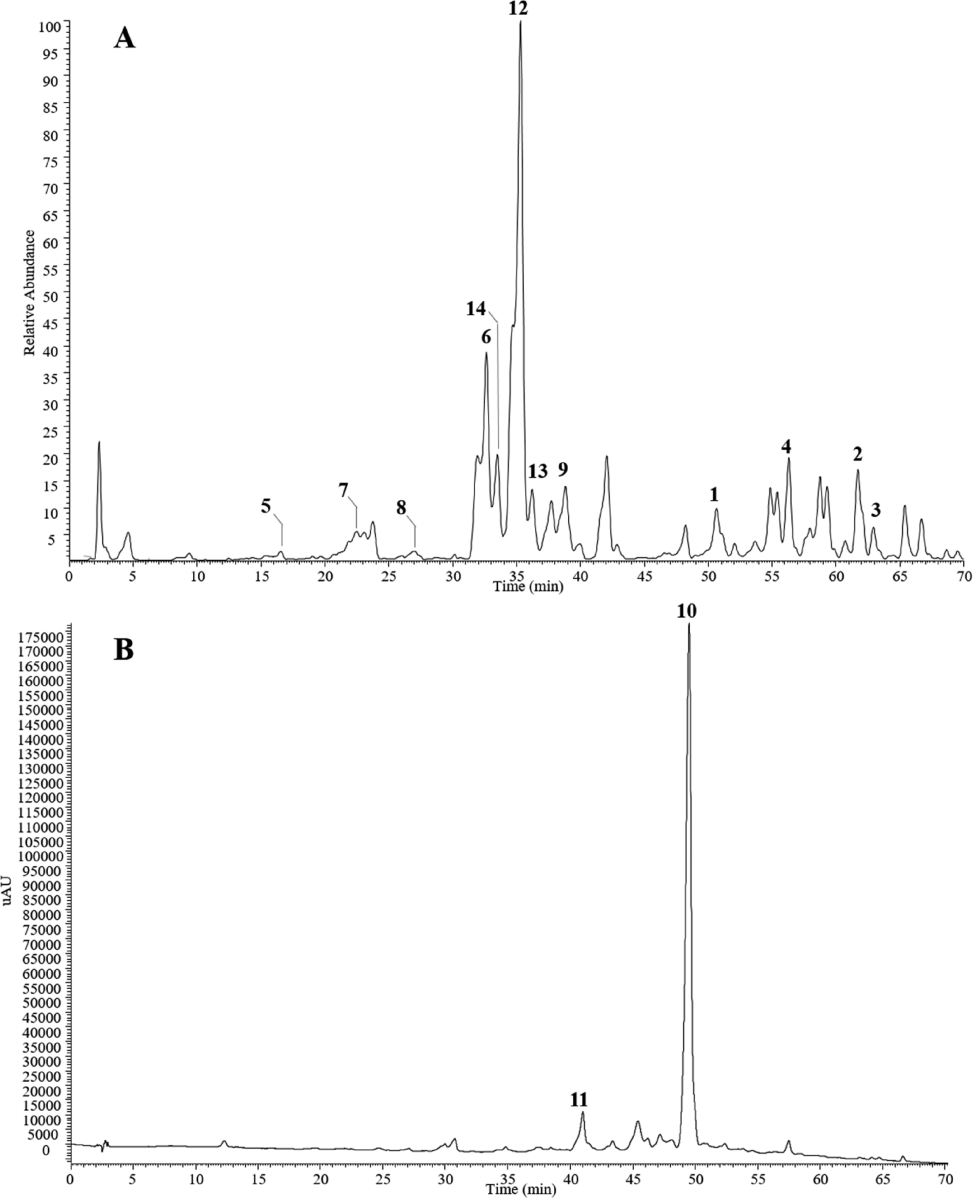
Chemical profiles of *Elaeagnus umbellata* fruit extracts. (A) HPLC-ESIMS of the *n*-BuOH extract registered in the negative ion mode. (B) HPLC/UV of the EtOAc extract registered at 350 nm. Peak numbers indicate the isolated compounds.

The fruits of *E. umbellata* were defatted with *n*-hexane and then extracted with MeOH. The MeOH extract was partitioned between EtOAc, *n*-BuOH, and H_2_O. The EtOAc and *n*-BuOH extracts, subjected to Isolera flash chromatography, Sephadex LH-20, and RP-HPLC separations, yielded in pure form five new (**1**–**5**) and nine known compounds (**6**–**14**). The chemical profiles of the EtOAc and *n*-BuOH extracts are shown in Figure 1.

The molecular formula of compound **1** was determined as C_65_H_104_O_32_ by HRESIMS, showing a sodium adduct ion at *m*/*z* 1419.6357 [M + Na]^+^. Analysis of the NMR data (Tables 1 and 2) confirmed the presence of 30 signals attributable to the aglycone moiety and 35 to the saccharide portion, establishing the presence of a bidesmosidic triterpenoid saponin. In the ESIMS data, fragments obtained in the negative and positive modes at *m*/*z* 1249 [M – H – 146]^−^, 1071 [M – H – 162 – 162]^−^, 1257 [M + Na – 162]^+^, and 1095 [M + Na – 162 – 162]^+^ suggested the presence of a triterpenoid saponin structure with at least one deoxyhexosyl and two hexosyl moieties. The ^1^H NMR data of **1** displayed seven methyl singlets at δ_H_ 0.87, 0.94, 0.99, 1.06, 1.09, 1.10, and 1.27, a hydroxymethine at δ 3.18 (dd, *J* = 11.5, 4.3 Hz), and six anomeric protons at δ 4.33 (d, *J* = 7.4 Hz), 4.44 (d, *J* = 6.8 Hz), 4.68 (d, *J* = 7.7 Hz), 4.77 (1H, d, *J* = 7.5 Hz), 5.45 (d, *J* = 8.0 Hz), and 5.61 (d, *J* = 1.8 Hz). The ^13^C NMR data suggested the structural features of 3-hydroxy-19-oxoolean-13-en-28 oic acid for the aglycone of compound **1** due to the presence of a hydroxymethine at δ_C_ 89.6 and an α,β-unsaturated carbonyl (δ_C_ 134.0, 153.3, and 211.0). This conclusion was supported by HMBC correlations between H_2_-16–C-14, H_2_-16–C-17, H_2_-16–C-18, H_2_-12–C-18, Me-29–C-19, Me-29–C-20, and Me-29–C-21.^21^ The structure of the sugar moieties was deduced using 1D TOCSY, COSY, HSQC, and HMBC experiments, leading to recognition of four β-glucopyranosyl, an α-arabinopyranosyl, and an α-rhamnopyranosyl moiety. The chemical shifts of one glucose anomeric proton at δ_H_ 5.45 and δ_C_ 95.9 suggested this sugar moiety to be involved in an ester linkage with the C-28 carboxylic acid group at δ_C_ 174.2, and the HMBC correlation peak between H-1_glc_–C-28 corroborated this substitution. Direct evidence of the sugar sequence at C-3 was derived from the HMBC correlations between H-1_ara_–C-3, H-1_glcI_–C-3_ara_, H-1_rha_–C-2_glcI_, H-1_glcII_–C-3_glcI_, and H-1_glcIII_–C-6_glcI_. The assignment of the sugar configuration was obtained through hydrolysis of **1** with 1 N HCl followed by GC analysis through a chiral phase column of the monosaccharides treated with 1-(trimethylsilyl)imidazole in pyridine. Thus, compound **1** was identified as 28-*O*-β-d-glucopyranosyl 3β-*O*-[β-d-glucopyranosyl-(1→6)]-[β-d-glucopyranosyl)-(1→3)]-[α-l-rhamnopyranosyl-(1→2)]-β-d-glucopyranosyl-(1→3)-α-l-arabinopyranosyl-19-oxoolean-13-en-28-oate.

**Table 1:** ^1^H and ^1^^3^C NMR Data for Aglycones of Compounds **1**, **2**, and **5**^a^

	**1**		**2**			**5**	
position	δ_H_	δ_C_	δ_H_	δ_C_	position	δ_H_	δ_C_
1a	1.77^b^	39.9	1.71^b^	39.0	2		174.0
1b	1.07, m		1.08^b^		3	5.91, s	126.6
2a	1.92^b^	27.0	2.05, m	26.5	4		140.0
2b	1.76^b^		1.74^b^		5	7.91, d (16.0)	133.8
3	3.18, dd (11.5, 4.3)	89.6	3.19, dd (12.0, 4.0)	89.5	6	6.25, d (16.0)	126.7
4		39.1		39.0	7		80.5
5	0.83, br d (11.7)	57.3	0.84, br d (11.5)	57.2	8		51.0
6a	1.64^b^	19.0	1.59^b^	19.0	9a	2.09, dd (14.0, 7.5)	39.1
6b					9b	1.93, dd (14.0, 11.0)	
7a	1.66^b^	34.3	1.68^b^	34.2	10	4.01, m	72.8
7b	1.41, m		1.41, m		11a	2.45, dd (14.4, 6.5)	39.0
8		43.0		40.0	11b	1.99, dd (14.4, 10.0)	
9	1.61^b^	52.2	1.71^b^	48.5	12		87.5
10		36.9		36.0	13	2.04, s	20.1
11a	1.61^b^	23.5	2.00^b^	24.1	14		178.0
11b			1.98^b^		15	1.12, s	14.0
12a	2.67, br dd (16.0, 12.0)	134.0	5.26, t (3.0)	129.4	16	1.40, s	18.2
12b	1.99, m				Glc-1	4.37, d (7.8)	103.0
13		153.3		133.0	2	3.16, br t (9.0)	74.6
14		45.0		41.8	3	3.26, t (9.5)	77.4
15a	1.92^b^	27.0	1.99^b^	28.0	4	3.30, t (9.5)	71.3
15b	1.76^b^		1.82, m		5	3.38, m	77.7
16a	2.15, ddd (14.3, 6.5, 3.8)	34.3	1.89^b^	27.0	6a	3.86, dd (12.0, 3.0)	62.2
16b	1.93^b^		1.62^b^		6b	3.68, dd (12.0, 5.0)	
17		53.1		50.0			
18		134.0	3.79, s	56.8			
19		211.0		216.5			
20		45.5		44.0			
21a	1.85, ddd (17.0, 14.3, 3.8)	36.6	1.93^b^	35.2			
21b	1.65^b^		1.77^b^				
22a	2.30, ddd (14.3, 6.6, 3.3)	32.0	2.26, ddd (15.0, 11.0, 4.0)	31.0			
22b	1.63^b^		1.80^b^				
23	1.06, s	28.0	1.07, s	28.1			
24	0.87, s	17.0	0.89, s	17.0			
25	0.99, s	17.0	1.00, s	16.0			
26	0.94, s	14.0	0.85, s	17.5			
27	1.27, s	21.0	0.97, s	23.0			
28		174.2		175.0			
29	1.09, s	26.5	1.22, s	27.0			
30	1.10, s	25.5	1.11, s	25.6			

a Spectra were recorded in methanol-*d*_4_ at 600 MHz (^1^H) and 150 MHz (^13^C). *J* values are in parentheses and reported in Hz; chemical shifts are given in ppm; assignments were confirmed by 1D-TOCSY, COSY, HSQC, and HMBC experiments.

b Overlapped signal.

**Table 2:** ^1^H and ^13^C NMR Data for Sugar Moieties of Compounds **1**–**3**^a^

	**1**		**2**		**3**	
position	δ_H_	δ_C_	δ_H_	δ_C_	δ_H_	δ_C_
ara-1 at C-3	4.44, d (6.8)	105.7	4.44, d (6.7)	105.8	4.44, d (6.8)	106.0
2	3.91, dd (9.0, 6.8)	75.0	3.93, dd (9.0, 6.7)	75.0	3.93, dd (9.0, 6.8)	75.1
3	3.95, dd (9.0, 2.0)	83.0	3.91, dd (9.0, 3.0)	82.7	3.91, dd (9.0, 2.5)	82.3
4	4.08, m	70.0	4.08, m	70.0	4.07, m	71.0
5a	3.88, dd (12.0, 2.0)	66.2	3.87, dd (12.0, 2.5)	66.0	3.90, dd (12.0, 2.0)	66.5
5b	3.65, dd (12.0, 4.0)		3.61, dd (12.0, 4.0)		3.62, dd (12.0, 4.5)	
glc I-1	4.68, d (7.7)	103.0	4.66, d (7.5)	102.8	4.68, d (8.0)	102.9
2	3.66^b^	77.0	3.64^b^	77.8	3.60, dd (9.5, 8.0)	77.8
3	3.65^b^	83.4	3.65^b^	83.3	3.66, t (9.5)	83.6
4	3.28, t (9.5)	71.0	3.40, t (9.5)	70.6	3.39, t (9.5)	71.0
5	3.55, m	76.7	3.33, m	77.9	3.31, m	78.0
6a	4.20, dd (12.0, 3.0)	70.0	3.88, dd (12.0, 3.5)	62.0	3.88, dd (12.0, 3.0)	62.4
6b	3.72, dd (12.0, 5.0)		3.72, dd (12.0, 5.0)		3.72, dd (12.0, 4.5)	
rha-1	5.61, d (1.8)	101.0	5.62, d (1.5)	100.7	5.64, d (1.8)	100.0
2	3.95, dd (3.0, 1.8)	72.0	3.96, dd (3.0, 1.5)	72.0	3.96, dd (3.0, 1.8)	72.1
3	3.73, dd (9.0, 3.0)	72.0	3.74, dd (9.0, 3.0)	71.8	3.72, dd (9.0, 3.0)	72.0
4	3.45, t (9.0)	74.0	3.45, t (9.0)	73.5	3.47, t (9.0)	73.0
5	4.07, m	70.0	4.10, m	70.0	4.09, m	70.5
6	1.26, d (6.5)	17.0	1.25, d (6.8)	17.4	1.28, d (6.5)	18.0
glc II-1	4.77, d (7.5)	105.7	4.78, d (7.5)	105.3	4.79, d (7.5)	105.6
2	3.38, dd (9.5, 7.5)	75.0	3.40^b^	75.0	3.39, dd (9.0, 7.5)	75.6
3	3.34, t (9.5)	78.0	3.42, t (9.0)	78.0	3.44, t (9.0)	78.0
4	3.40, t (9.5)	70.0	3.40^b^	71.0	3.43, t (9.0)	71.0
5	3.36, m	77.0	3.33, m	77.9	3.31, m	78.3
6a	3.93, dd (12.0, 3.0)	62.0	3.90, dd (12.0, 2.5)	62.1	3.93, dd (12.0, 3.0)	62.1
6b	3.75, dd (12.0, 4.5)		3.80, dd (12.0, 4.5)		3.77, dd (12.0, 5.0)	
glc III-1	4.33, d (7.4)	104.9				
2	3.23, dd (9.0, 7.4)	75.0				
3	3.35, t (9.0)	77.0				
4	3.28, t (9.0)	71.0				
5	3.41, m	77.0				
6a	3.90, dd (12.0, 3.0)	62.0				
6b	3.69, dd (12.0, 5.0)					
glc-1 at C-28	5.45, d (8.0)	95.9	5.46, d (8.0)	95.7		
2	3.29, dd (9.5, 8.0)	75.0	3.35, dd (9.5, 8.0)	73.4		
3	3.41^b^	77.7	3.33, t (9.5)	77.9		
4	3.40^b^	70.0	3.40^b^	70.6		
5	3.36, m	77.0	3.40^b^	78.0		
6a	3.83, dd (12.0, 3.0)	62.0	3.86, dd (12.0, 2.5)	62.0		
6b	3.70, dd (12.0, 4.5)		3.72, dd (12.0, 5.0)			
rha-1 at C-28					6.00, d (1.8)	95.5
2					3.96, dd (3.0, 1.8)	72.1
3					3.72, dd (9.5, 3.0)	72.0
4					3.44, t (9.5)	73.0
5					4.09, m	70.5
6					1.24, d (6.0)	18.0

a Spectra were recorded in methanol-*d*_4_ at 600 MHz (^1^H) and 150 MHz (^13^C). *J* values are in parentheses and reported in Hz; chemical shifts are given in ppm; assignments were confirmed by COSY, 1D-TOCSY, HSQC, and HMBC experiments.

b Overlapped signal.

Compound **2** showed a sodium adduct ion at *m*/*z* 1257.5826 [M + Na]^+^, differing by 162 Da from **1**. In the ESIMS fragments obtained in the positive ion mode at *m*/*z* 1095 [M + Na – 162]^+^, 1111 [M + Na – 146]^+^, 933 [M + Na – 162 – 162]^+^, 625 [M + Na – 162 – 162 – 146 – 162]^+^, and 447 [M + Na – 162 – 162 – 146 – 162 – 132 – 46]^+^ were consistent with the presence of a triterpenoid saponin with five sugar moieties comprising three hexosyl, one deoxyhexosyl, and one pentosyl unit. Analysis of the NMR data (Table 1) for the aglycone moiety showed the presence of nine methylenes, seven methyls, four methines (one oxygenated), an sp^2^ methine, seven quaternary carbons (one sp^2^), a keto, and a hydroxy carbonyl carbon. The MS and NMR data displayed for the aglycone moiety eight indices of hydrogen deficiency. The ^1^H NMR spectrum showed, in addition to signals corresponding to seven methyls, a hydroxymethine at δ 3.19 (dd, *J* = 12.0, 4.0 Hz) and an olefinic proton at δ 5.26 (t, *J* = 3.0 Hz). The HMBC spectrum showed correlations between Me-29 (δ 1.22) and C-19, C-20, and C-21 and between H-18 (δ 3.79) and C-12, C-16, C-18, C-19, and C-20. The spectroscopic features (Table 1) were in agreement with a 19-oxooleanolic acid as the aglycone of compound **2**. This triterpenoid aglycone structure is described here for the first time. Comparison of the spectroscopic data of the sugar portion of compounds **2** and **1** (Table 2) showed structural similarities: in particular, the saccharide chain of **2** differed from that of **1** only in the absence of the β-glucopyranosyl moiety linked at C-6 of glc I. The configuration of the saccharide units was assigned as reported for **1**. Thus, compound **2** was characterized as 28-*O*-β-d-glucopyranosyl 3β-*O*-β-d-glucopyranosyl-(1→3)-β-d-glucopyranosyl-(1→2)-[α-l-rhamnopyranosyl]-(1→3)-α-l-arabinopyranosyl-19-oxooleanolate.

Compound **3** (C_59_H_94_O_26_), isolated in a small amount, exhibited a sodium adduct ion at *m*/*z* 1241.5990 [M + Na]^+^, as well as a fragment peak at *m*/*z* 1095.6798 [M + Na – 146]^+^ corresponding to the loss of a deoxyhexosyl unit. Analysis of its NMR spectroscopic data (Table 2) and comparison with those of saponin **2** showed that the aglycone moiety and the sugar chain at C-3 of **3** were the same as those of **2**, while the sugar moiety at C-28 was different. The presence of an anomeric proton at δ_H_ 6.00 and at δ_C_ 95.5 belonging to a terminal rhamnopyranosyl moiety suggested this sugar to be linked at C-28. This assumption was substantiated by the HMBC correlation between H-1_rha_ (δ_H_ 6.00) and C-28 (δ_C_ 175.0). The configuration of the saccharide units was determined as reported for **1**. The structure of **3** was thus established as 28-*O*-α-l-rhamnopyranosyl 3β-*O*-d-glucopyranosyl-(1→3)-β-d-glucopyranosyl-(1→2)-[α-l-rhamnopyranosyl]-(1→3)-α-l-arabinopyranosyl-19-oxooleanolate.

Compound **4** was isolated in a trace amount. Its HRESIMS data in the positive ion mode showed an [M + Na]^+^ ion at *m*/*z* 1419.6390, corresponding to a molecular formula of C_65_H_104_O_32_, and hence was shown to be an isomer of **1**. The HRESIMS/MS data revealed the presence of fragments at *m*/*z* 1257.7781 [M + Na – 162]^+^ and 1095.6188 [M + Na – 162 – 162]^+^ due to the subsequent loss of two hexosyl moieties. Comparison of its NMR spectroscopic data with those of saponins **1**–**3** showed **4** to have the same aglycone as **2**, while its saccharidic chains at C-3 and C-28 were identical to those of **1**. The aglycone moiety of compound **1** could be an artifact of **4**. The double-bond rearrangement could be due to the formation of a conjugated α,β-unsaturated carbonyl system. Therefore, the structure 28-*O*-β-d-glucopyranosyl 3-*O*-[β-d-glucopyranosyl-(1→6)]-[β-d-glucopyranosyl)-(1→3)]-[α-l-rhamnopyranosyl-(1→2)]-β-d-glucopyranosyl-(1→3)-α-l-arabinopyranosyl-19-oxooleanolate was assigned to compound **4**.

Compound **5** had the molecular formula C_21_H_30_O_11_ as determined by HRESIMS (*m*/*z* 481.1666 [M + Na]^+^). Its ESIMS data recorded in the negative ion mode displayed fragments at *m*/*z* 413 [M – H – 44]^−^, 295 [M – H – 162]^−^, 251 [M – H – 162 – 44]^−^, 233 [M – H – 162 – 18 – 44]^−^, and 189 [M – H – 162 – 18 – 44 – 44]^−^_,_ due to the subsequent loss of CO_2_, a hexosyl unit, H_2_O, and a second CO_2_ molecule. These data supported the presence of two carboxylic groups in the structure of **5**. The ^13^C NMR data of **5** (Table 1) showed 21 signals, of which 15 were assigned to a sesquiterpenoid moiety and six to the sugar portion. The signals of the aglycone moiety were sorted into three methyls, two methylenes, three sp^2^ methines, a hydroxymethine, two quaternary carbons, two oxygenate tertiary carbons, a hydroxycarbonyl carbon, and an ester carbonyl. The ^1^H NMR data (Table 1) displayed three methyl singlets at δ 1.12, 1.40, and 2.04, the latter having a chemical shift typical of an allylic methyl, together with signals of three olefinic protons at δ_H_ 5.91 (s), 6.25 (d, *J* = 16.0 Hz), and 7.91 (d, *J* = 16.0 Hz), a hydroxymethine (δ 4.01, m), and an anomeric proton (δ 4.37, d, *J* = 7.8 Hz). The COSY experiment indicated two spin systems for the aglycone corresponding to the sequence −CH_2_–CHOH–CH_2_– and −CH=CH–C–CH–, respectively. The HSQC experiment allowed the assignments of all protons linked to the respective carbons, to identify the β-glucopyranosyl moiety, while the HMBC spectrum was crucial to identify the aglycone as amygdalactone.^22^ The HMBC correlation between H-1_glc_ at δ_H_ 4.37 and C-10 at δ_C_ 72.8 confirmed the glucosyl substitution site. On the basis of these data, compound **5** was identified as 10-*O*-β-d-glucopyranosylamygdalactone (**5**). The aglycone amygdalactone was isolated before only from *Prunus amygdalus*(22) and *Cinnamomum cassia*.^23^

Compounds **6**–**14** were also purified and characterized as kaempferol 3-*O*-β-d-glucopyranosyl-(1→2)-β-d-galactopyranoside-7-*O*-α-l-rhamnopyranoside (**6**),^24^ 2-hydroxynaringenin 7-*O*-β-d-glucopyranoside (**7**),^25^ 1-*O*-(*trans*-sinapoyl)-β-d-glucopyranoside (**8**),^26^ isorhamnetin 3-*O*-α-l-xylopyranosyl-(1→2)-β-d-glucopyranoside (**9**),^27^ tiliroside (**10**),^28^ kaempferol 3-*O*-β-d-glucopyranoside (**11**),^29^ quercetin 3-*O*-α-l-xylopyranosyl-(1→2)-β-d-galactopyranoside (**12**),^30^ kaempferol 3-*O*-β-d-glucopyranosyl-(1→2)-β-d-galactopyranoside (**13**),^31^ and quercetin 3-*O*-β-d-glucopyranosyl-(1→2)-β-d-galactopyranoside-7-*O*-α-l-rhamnopyranoside (**14**),^27^ by comparison of their NMR and MS data to literature data.

**Figure 2:**
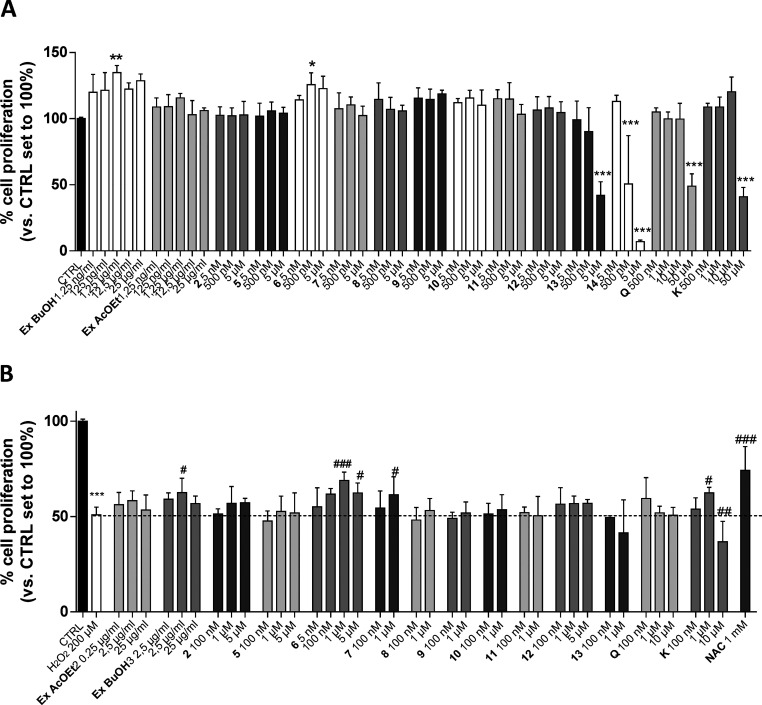
Protective effects of *Elaeagnus umbellata* extracts and isolated compounds on hGF. Human GF cells were treated with different concentrations of Ex EtOAc, Ex BuOH, compounds **2**, **5**, and **6**–**14**, quercetin (**Q**), kaempferol (**K**), or NAC (1 mM) in the absence (A) or presence (B) of H_2_O_2_ (200 μM) for 72 h in complete medium. In the end, the MTS assay was performed. Data are expressed as percentage of cell proliferation compared to the control, which was set to 100%. Each bar represents the mean ± SD of three replicates from two independent experiments. The significance of the differences was determined by one-way ANOVA, which was followed by Bonferroni’s post-test: *p < 0.05, **p < 0.01, ***p < 0.001, vs CTRL; ^#^*p* < 0.05, ^##^*p* < 0.01, ^###^*p* < 0.001, vs H_2_O_2_.

Compound **2**, as a representative saponin, compounds **5**–**14**, and the EtOAc (Ex EtOAc) and *n*-BuOH (Ex BuOH) extracts were screened for their potential proliferative effects on human gingival fibroblast (hGF) using the MTS assay (Figure 2A). Among the compounds, **13** and **14** produced a significant decrease in cell proliferation when used at micromolar concentrations. Conversely, Ex BuOH and compound **6** (1 μM) were able to significantly increase the hGF proliferation to 122.3 ± 4.8% and 125.6 ± 9.1%, respectively, showing a beneficial effect of compound **6** on hGF. Challenging the fibroblast with low concentrations of quercetin (**Q**) and kaempferol (**K**) did not affect the proliferation of hGF. These data are in accordance with the ability of kaempferol glycosides to increase keratinocyte proliferation more than kaempferol itself.^32^ Conversely, a high concentration (50 μM) of **Q** and **K** caused a significant decrease in cell proliferation. The treatment with a high concentration of H_2_O_2_ (millimolar range) causes cell death in different cellular models. However, the use of a micromolar concentration of H_2_O_2_ for a prolonged period induces DNA damage, cell cycle arrest, and the expression of senescence-associate protein (p53, p21, and p16).^33^ Herein, we used a low concentration of H_2_O_2_ (200 μM) that better reproduces the effects of ROS in periodontitis and mucositis. The ability of all the tested compounds to counteract the decrease of proliferation induced by H_2_O_2_ was evaluated using the MTS assay (Figure 2B). Challenging hGF cells with H_2_O_2_ for 72 h significantly decreased the proliferation (50.9 ± 4.0%, *p* < 0.001 vs CTRL), and these effects were counteracted by *N*-acetylcysteine (NAC) tested as reference antioxidant molecule (74.1 ± 12.6%, *p* < 0.001 vs CTRL).^34^ Interestingly, Ex BuOH counteracted the decrease of cell proliferation (62.5 ± 7.5%, *p* < 0.05 vs CTRL), and compound **6** produced a more robust effect (68.8 ± 4.4%, *p* < 0.01 vs CTRL).

**Figure 3:**
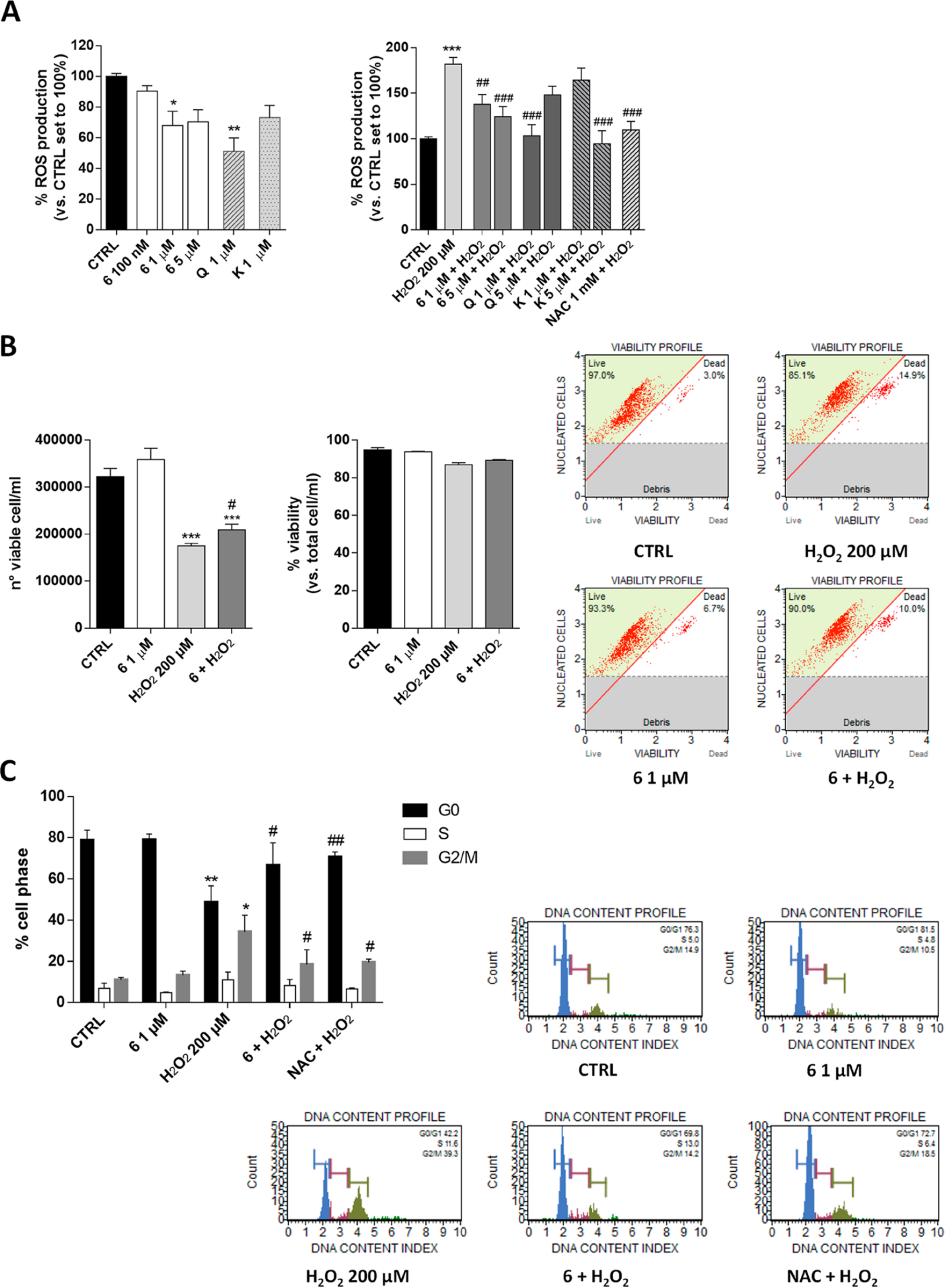
Effect of compound **6** on ROS production, cell death, and cell cycle progression. hGF cells were treated with different concentrations of compound **6**, quercetin (**Q**), kaempferol (**K**), or NAC as indicated, alone or in the presence of H_2_O_2_ (200 μM) for 72 h in complete medium. The ROS levels were evaluated by H_2_DCFDA (A). Data are expressed as the percentage of ROS with respect to the amount in the CTRL set to 100%. Each bar represents the mean ± SD of three replicates from two independent experiments. (B) Flow cytometry analysis of cell death was performed as described in the Experimental Section. Data are reported as the number of cells/mL and as percentage of cell death. Each bar represents the mean ± SD of two replicates from two independent experiments. Representative histograms are shown. (C) Flow cytometry analysis of the cell cycle was performed as described in the Experimental Section. Data are reported as the percentage of cells in each cell phase. Each bar represents the mean ± SD of two replicates from two independent experiments. Representative histograms are shown. The significance of the differences was determined by one-way ANOVA, which was followed by Bonferroni’s post-test: *p < 0.05, **p < 0.01, ***p < 0.001, vs CTRL; ^#^*p* < 0.05, ^##^*p* < 0.01, ^###^*p* < 0.001 vs H_2_O_2_.

Successively, we extensively investigated the effects of compound **6** on hGF as a new potential biological activity of a rare flavonoid glycoside. The correct balance of intracellular ROS level positively regulates physiological functions, including signal transduction, gene expression, and proliferation, favoring adaptive responses and longevity. In contrast, the uncontrolled production of ROS induces ineffective adaptive responses, contributing to aging phenomena.^35^ In this respect, the antioxidant ability of compound **6** was investigated in the absence or presence of H_2_O_2_ using H_2_DCFDA, which is the reduced form of fluorescein used as an indicator of intracellular ROS levels (Figure 3A). Compound **6** alone decreased the intracellular ROS concentration (*p* < 0.05). Despite the fact that this effect was similar to that obtained with **K**, a low concentration of **Q** produced a more robust effect, in accordance with its antioxidant effects reported in other cell lines.^36^ Interestingly, compound **6** significantly counteracted the ROS production induced by H_2_O_2_ (181.9 ± 7.4% H_2_O_2_, 124.2 ± 11.3% **6**, *p* < 0.001).^37,38^ This effect was similar to that obtained for NAC (109.7 ± 9.4%, *p* < 0.001 vs H_2_O_2_). Overall, these results indicated that the flavonol derivate of *E. umbellata* slightly alters the balance of intracellular ROS in physiological conditions, but could effectively counteract the stress effects produced by the external stimuli.

The modification of cell proliferation could be due to the induction of cell death or a blockade of the cell cycle. In order to extensively investigate the well-being activity of compound **6**, a live/death cell count assay was performed (Figure 3B). The results demonstrated that treatment with H_2_O_2_ did not induce significant cell death, in accordance with the use of a low concentration. Similarly, the treatment with compound **6** alone or in combination with H_2_O_2_ did not alter the number of dead cells. Compound **6** alone did not produce a significant increase in cell number (322 400 ± 39431, CTRL; 358 800 ± 53942); however, it significantly counteracted the decrease of cell number induced by H_2_O_2_ (175 200 ± 11 777, H_2_O_2_; 209 500 ± 23502, **6** + H_2_O_2_; *p* < 0.05), in accordance with the results obtained for cell proliferation (Figure 2). Next, a flow cytometry analysis was performed to investigate the cell cycle progression (Figure 3C). hGF cells in the absence of H_2_O_2_ treatment showed a typical cell cycle distribution with most cells in G0/G1 phase (78.7 ± 4.9%). The treatment with H_2_O_2_ significantly increased the number of cells in the S and G2/M phases (from 11.1 ± 1.2% to 34.3 ± 8.1%; *p* < 0.05), as well as decreased the number of cells in the G0/G1 phase (48.6 ± 7.9%; *p* < 0.001), in accordance with the reported ability of ROS to produce a G2/M cell cycle blockade.^39^ This modification was almost completely counteracted by NAC (70.6 ± 2.5%, G0/G1; *p* < 0.01; 19.5 ± 1.2%, G2/M; *p* < 0.05). Interestingly, compound **6** counteracted the cell cycle blockade, increased the cells in the G0/G1 phase (66.6 ± 10.9%; *p* < 0.05), and reduced those in the G2/M phase (18.5 ± 7.1%; *p* < 0.05).

**Figure 4:**
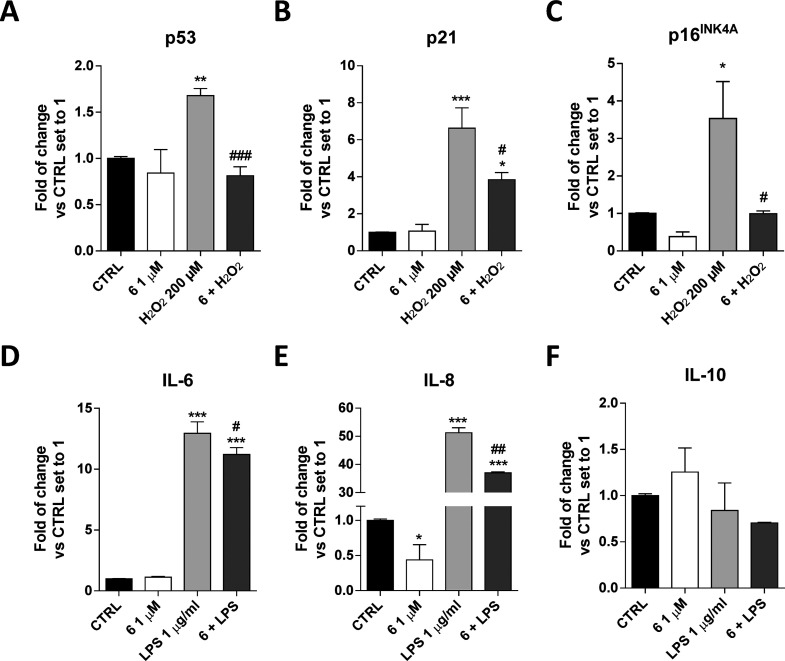
Compound **6** modulation of senescence-associated and cytokine gene expression in hGF. hGF cells were treated with different concentrations of compound **6** alone or in the presence of H_2_O_2_ (200 μM) or LPS (1 μg/mL) for 72 h in complete medium. Then, the mRNA levels of p53 (A), p21 (B), p16^INK4A^ (C), IL-6 (D), IL-8 (E), and IL-10 (F) were quantified using real-time RT-PCR analysis. Data are expressed as the fold change versus the CTRL, which was set to 1, and are presented as the mean values ± SD of two independent experiments performed in duplicate. The significance of the differences was determined by one-way ANOVA, which was followed by Bonferroni’s post-test: *p < 0.05, **p < 0.01, ***p < 0.001 vs CTRL; ^#^*p* < 0.05, ^##^*p* < 0.01, ^###^*p* < 0.001 vs H_2_O_2_ or LPS.

So far, oxidative stress has been associated with senescence and growth arrest, and it has been shown that these phenomena depend functionally on the expression of different proteins such as p16^INK4^, the cyclin-dependent kinase (CDK) inhibitor p21, and tumor suppressor p53.^40^ In this respect, the gene expression of these proteins was evaluated by means of real-time reverse transcription (RT)-PCR (Figure 4A–C). Compound **6** “per se” did not alter the p21 and p53 transcription and produced only a slight decrease of p16 in accordance with its ability to promote the hGF cell proliferation. The oxidative stress caused a significant increase of p53, p21, and p16 gene expression, in accordance with the H_2_O_2_-induced cell blockage. Compound **6** counteracted the effects of H_2_O_2_, decreasing the transcription of all the proteins.

Periodontitis and mucositis are also characterized by the presence of high levels of pro-inflammatory cytokines that are released in response to extracellular stimuli (e.g., bacteria) by different types of cells such as fibroblasts.^41^ Thus, to further characterize the beneficial activity of compound **6** on gingival cells, the gene expression of pro-inflammatory (interleukin-6, IL6, and interleukin-8, IL-8) and anti-inflammatory (interleukin-10, IL-10) cytokines was evaluated using a real-time RT-PCR analysis (Figure 4D–F). Challenging cells with LPS (1 μg/mL) promoted the transcription of IL-6 and IL-8 and the decrease of IL-10. Compound **6** restored the balance of the released cytokines in favor of a lower inflammatory status; in fact, it reduced the expression of IL-6 and IL-8 and increased the expression of IL-10.

Compound **6** has recently been reported as a dipeptidyl peptidase IV (DPP-IV) inhibitor,^42^ and different kaempferol glycosides have been reported to increase keratinocyte proliferation^32^ and protect red blood cells and neutrophils from DNA damage;^36,43^ however, its effect on gingival tissue has not yet been reported.

Compound **6** counteracted the negative effects of oxidative stress and decreased the gene expression of pro-inflammatory cytokines. Poor absorption of some flavonoids and flavonoid glycosides is a major limitation for use of flavonoids as systemic nutraceuticals.^44^ However, the ability of a flavonoid to act from the extracellular compartment modifying the membrane composition and interacting with different membrane receptors has recently been reported.^45^ The exact mechanism of action of compound **6** is still unclear; however we could speculate that the glycoside or its metabolites could interact with extracellular cell components, modifying the hGF oxidative state and decreasing the inflammatory status. In conclusion, these results highlight a new property of a flavonoid glycoside as a possible local protective agent against oxidative stress and inflammatory stimuli in gingival tissue.

## Experimental Section

### General Experimental Procedures

Optical rotations were measured by an Atago AP-300 digital polarimeter with a 1 dm microcell and a sodium lamp (589 nm). NMR experiments were recorded on a Bruker DRX-600 spectrometer at 300 K (Bruker BioSpin, Germany), acquiring the spectra in methanol-*d*_4_.^46^ HRESIMS data were obtained in the positive and negative ion mode on an LTQ Orbitrap XL mass spectrometer (Thermo Fisher Scientific) and Q-TOF Premier spectrometer equipped with a nanospray ion source (Waters, USA). ESIMS data were acquired on an LCQ Advantage ThermoFinnigan spectrometer (ThermoFinnigan, USA), equipped with Xcalibur software. LC-PDA/UVvis/ESIMS/MS analyses were performed on a Synergi Fusion-RP column (4.6 × 150 mm, 4 μm, flow rate 0.8 mL/min, Phenomenex, Italy), eluting with a mixture of MeOH (solvent A) and formic acid in water 0.1% v/v (solvent B) and using a linear gradient of increasing 5% to 75% MeOH within 70 min. MS parameters were optimized as previously reported.^47^ Columns chromatography was performed over Sephadex LH-20 and an Isolera Biotage flash purification system (flash silica gel 60 SNAP cartridge). HPLC separations were carried out using a Shimadzu LC-8A series pumping system equipped with a Shimadzu RID-10A refractive index detector and Shimadzu injector (Shimadzu Corporation, Japan) on a C_18_ μ-Bondapak column (30 cm × 7.8 mm, 10 mm, flow rate 2.0 mL/min). TLC separations were conducted using silica gel 60 F_254_ (0.20 mm thickness) plates (Merck, Germany) and Ce(SO_4_)_2_–H_2_SO_4_ as spray reagent (Sigma-Aldrich, Italy). GC analysis was performed using a Dani GC 1000 instrument on a l-CP-Chirasil-Val column (0.32 mm × 25 m), working with the following temperature program: 100 °C for 1 min, ramp of 5 °C/min up to 180 °C; injector and detector temperature 200 °C; carrier gas N_2_ (2 mL/min); detector dual FID; split ratio 1:30; injection 5 μL.

### Plant Material

The ripe fruits of *Elaeagnus umbellata* Thunb. were collected in October 2016 in Livorno (Italy) and identified by Prof. Fabiano Camangi. A voucher specimen (N.A. 5472 *Elaeagnus umbellata*/026677) was deposited at Herbarium Horti Botanici Pisani (Pisa, Italy).

### Extraction and Isolation

Dried and powdered whole berries of *E. umbellata* (400.0 g) were defatted with *n*-hexane and extracted for 48 h with MeOH by exhaustive maceration (3 × 2.5 L), to give 137.7 g of extract. Dried MeOH extract was dissolved in water and partitioned with EtOAc and *n*-BuOH, successively, to yield 5.0 and 1.8 g of the respective residues. The *n*-BuOH extract was subjected to Sephadex LH-20 column chromatography (3 × 70 cm) using MeOH as eluent (flow rate 0.8 mL/min), collecting fractions of 8 mL that were grouped by thin-layer chromatography (TLC) into 10 major fractions (A_1_–J_1_). Fractions A_1_ (25.5 mg), B_1_ (35.5 mg), and C_1_ (65.0 mg) were separately subjected to RP-HPLC with MeOH–H_2_O (3:2) to yield compounds **4** (0.8 mg, *t*_R_ 16 min) and **1** (1.8 mg, *t*_R_ 41 min) from fraction A_1_, compounds **2** (0.7 mg, *t*_R_ 35 min) and **1** (1.2 mg, *t*_R_ 41 min) from fraction B_1_, and compounds **2** (1.8 mg, *t*_R_ 35 min) and **3** (1.1 mg, *t*_R_ 55 min) from fraction C_1_. Fractions E_1_ (115.0 mg) and G_1_ (104.0 mg) were individually chromatographed by RP-HPLC with MeOH–H_2_O (3.5:6.5) to give compound **5** (1.9 mg, *t*_R_ 8 min) from fraction E_1_ and compounds **8** (1.0 mg, *t*_R_ 12 min), **7** (3.2 mg, *t*_R_ 16 min), **14** (4.5 mg, *t*_R_ 31 min), and **13** (3.7 mg, *t*_R_ 57 min) from fraction G_1_. Fractions F_1_ (63.2 mg) and I_1_ (25.0 mg) were separately purified by RP-HPLC with MeOH–H_2_O (2:3) to afford compound **6** (3.8 mg, *t*_R_ 16 min) from fraction F_1_ and compound **9** (1.8 mg, *t*_R_ 44 min) from fraction I_1_. Fraction J_1_ (67.1 mg) contained pure compound **12**.

The EtOAc fraction was subjected to flash silica gel CC using an Isolera Biotage (SNAP 340 g column, flow rate 90 mL/min), eluting with CHCl_3_, followed by increasing concentrations of MeOH in CHCl_3_ (between 1% and 100%), collecting fractions of 27 mL, which were grouped by TLC into four major fractions (A_2_–D_2_). Fractions B_2_ (69.0 mg) and C_2_ (105.6 mg) were subjected to RP-HPLC with MeOH–H_2_O (5.5:4.5) to yield compound **10** (1.7 mg, *t*_R_ 31 min) from fraction B_2_ and compounds **11** (2.8 mg, *t*_R_ 17 min) and **10** (11.4 mg, *t*_R_ 37 min) from fraction C_2_.

#### Compound (**1**):

amorphous powder; [α]_D_^25^ −8 (*c* 0.1, MeOH); ^1^H and ^13^C NMR data of the aglycone moiety, see Table 1; ^1^H and ^13^C NMR of the sugar moieties, see Table 2; ESIMS *m*/*z* 1395 [M – H]^−^, 1249 [M – H – 146]^−^, 1071 [M – H – 162 – 162]^−^, 1419 [M + Na]^+^, 1257 [M + Na – 162]^+^, 1095 [M + Na – 162 – 162]^+^; HRESIMS *m*/*z* 1419.6357 [M + Na]^+^, 1257.5852 [M + Na – 162]^+^ (calcd for C_65_H_104_O_32_Na 1419.6403).

#### Compound (**2**):

amorphous powder; [α]_D_^25^ −10.1 (*c* 0.1, MeOH); ^1^H and ^13^C NMR data of the aglycone moiety, see Table 1; ^1^H and ^13^C NMR of the sugar moieties, see Table 2; ESIMS *m*/*z* 1257 [M + Na]^+^, 1095 [M + Na – 162]^+^, 1111 [M + Na – 146]^+^, 933 [M + Na – 162 – 162]^+^, 625 [M + Na – 162 – 162 – 146 – 162]^+^, 447 [M + Na – 162 – 162 – 146 – 162 – 132 – 46]^+^; HRESIMS *m*/*z* 1257.5826 [M + Na]^+^, 1095.6554 [M + Na – 162]^+^ (calcd for C_59_H_94_O_27_Na 1257.5875).

#### Compound (**3**):

amorphous powder; [α]_D_^25^ −21 (*c* 0.1, MeOH); ^1^H and ^13^C NMR data of the aglycone moiety were superimposable to those reported for **2**; ^1^H and ^13^C NMR of the sugar moieties, see Table 2; HRESIMS *m*/*z* 1241.5990 [M + Na]^+^, 1095.6798 [M + Na – 146]^+^ (calcd for C_59_H_94_O_26_Na 1241.5926).

#### Compound (**4**):

amorphous powder; [α]_D_^25^ −7 (*c* 0.1, MeOH); ^1^H and ^13^C NMR data of the aglycone moiety were superimposable to those reported for **2**; ^1^H and ^13^C NMR of the sugar moieties were superimposable to those reported for **1**; HRESIMS *m*/*z* 1419.6390 [M + Na]^+^, 1257.7781 [M + Na – 162]^+^, 1095.6188 [M + Na – 162 – 162]^+^ (calcd for C_65_H_104_O_32_Na 1419.6403).

#### Compound (**5**):

amorphous powder; [α]_D_^25^ −25 (*c* 0.1, MeOH); ^1^H and ^13^C NMR, see Tables 1 and 2; ESIMS *m*/*z* 457 [M – H]^−^, 413 [M – H – 44]^−^, 295 [M – H – 162]^−^, 277 [M – H – 180]^−^, 251 [M – H – 162 – 44]^−^, 233 [M – H – 162 – 18 – 44]^−^, 189 [M – H – 162 – 18 – 44 – 44]^−^_,_ 481 [M + Na]^+^, 463 [M + Na – 18]^+^, 437 [M + Na – 44]^+^; HRESIMS *m*/*z* 481.1666 [M + Na]^+^ (calcd for C_21_H_30_O_11_Na 481.1680).

### Acid Hydrolysis of Compounds **1**–**5**

Acid hydrolysis of compounds **1**–**5** was performed as reported previously.^39^d-Glucose, l-rhamnose, and l-arabinose were identified as the sugar unit in each case by comparison with the retention times of authentic samples.

### Cell Culture

Human gingival fibroblast cells were purchased from CLS Cell Line Service GmbH (Germany), lot. number 300703-1541SF. Cells were maintained in DMEM-F12 supplemented with HEPES, 5% FBS, 2 mM l-glutamine, 100 U/mL penicillin, and 100 mg/mL streptomycin at 37 °C in 5% CO_2_. Cells were used between passage 4 and 7.

### Cell Proliferation Assay

hGF cells were seeded in 96-well microplates (1500 cells/well) in complete medium. After 24 h the medium was changed to noncomplete medium and cells were starved for 6 h. Then, cells were treated with test extracts and compounds at different concentrations (5 nM to 50 μM) in the absence or in the presence of H_2_O_2_ (200 μM) for 72 h. Extracts and compounds were solubilized in DMSO, and the final concentration of DMSO was 0.5%. Cell proliferation was evaluated using the MTS assay (CellTiter 96 AQueous One Solution cell proliferation assay kit; Promega) according to the manufacturer’s instruction. The absorbance at 490 nm was measured with an automated plate reader (EnSight, PerkinElmer).

### ROS Production

The intracellular ROS level was determined using the fluorogenic probe DCFH_2_-DA (Molecular Probes, Invitrogen) as was previously reported.^48^ Briefly, hGF cells were seeded in a 96-well microplate (5000 cells/well) and treated with compound **6**, **Q**, or **K** (1–5 μM) in the absence or in the presence of H_2_O_2_ (200 μM) for 72 h. Then, cells were incubated in phosphate-buffered saline (PBS)–glucose containing 50 μM DCFH_2_-DA for 30 min. The medium was removed and replaced with PBS/glucose. The fluorescence intensity of DCF was measured with an automated plate reader (EnSight, PerkinElmer), with wavelengths of 485 nm (excitation) and 520 nm (emission). The cells were fixed with 3% paraformaldehyde for 20 min, washed with PBS, and incubated with crystal violet for 20 min. Finally, cells were washed, a solution of sodium dodecyl sulfate (1%) was added for 1 h, and the absorbance at 595 nm was quantified. The fluorescence intensity was normalized to the absorbance at 595 nm and related to the control value.

### hGF Live/Dead Cell Count and Cell Cycle Analyses

For cell live/dead cell count hGF were seeded in a six-well microplate (80 000 cells/well) and were treated in complete medium with compound **6** (1–5 μM) in the absence or in the presence of H_2_O_2_ (200 μM) for 72 h. The number of live cells and the percentage of dead cells were quantified and analyzed by Muse Cell Analyzer (Merck KGaA, Darmstadt, Germany), according to the manufacturer’s instructions (MCH100102 Muse Count & Viability assay kit, Merck Millipore). For the cell cycle analysis, hGF were seeded and treated as above. The quantification of the percentage of cells in the different cell phases was performed using the Muse Cell Analyzer (MCH100106 Muse cell cycle assay kit, Merck Millipore) as previously reported.^49^

### Real-Time RT-PCR Analysis

The gene expression in hGF was quantified by performing a real-time RT-PCR analysis. Briefly, hGF (3500 cells/cm^2^) were treated in complete medium with compound **6** (1–5 μM) in the absence or in the presence of H_2_O_2_ (200 μM) or LPS (1 μg/mL) for 72 h. The cells were collected, and the total RNA was extracted using the Rneasy mini kit (Qiagen, Hilden, Germany) according to the manufacturer’s instructions. The purity of the RNA samples was determined by measuring the absorbance at 260/280 nm. mRNA (500 ng) was retrotranscribed to single-strand cDNA using the i-Script cDNA synthesis kit (BioRad) following the manufacturer’s instructions. Real-time RT-PCR reactions were performed with Fluocycle II SYBR in the presence of 50 ng of cDNA and 200 nM forward and reverse primers.^48,50^ The primers were designed to span intron/exon boundaries to exclude genomic DNA impurity, and β-actin was used as the housekeeping gene. p53 FOR: 5′-CTTTGAGGTGCGTGTTTGTG-3′ REV: 5′-GTGGTTTCTTCTTTGGCTGG-3′ (161 bp); p21 FOR: 5′-TGCCGAAGTCAGTTCCTTG-3′ REV: 5′-CATGGGTTCTGACGGACATC-3′ (134 bp); p16^INK4A^ (CDKN2A) FOR: 5′-GACCCCGCCACTCTCACC-3′ REV: 5′-CCTGTAGGACCTTCGGTGACTGA-3′ (318 bp); IL-6 FOR: 5′-TCCTCGACGGCATCTCA-3′ REV: 5′-TTTTCACCAGGCAAGTCTCCT-3′ (165 bp); IL-8 FOR: 5′-AAGAGAGCTCTGTCTGGACC-3′ REV: 5′-GATATTCTCTTGGCCCTTGG-3′ (408 bp); IL-10 FOR: 5′-CAAGCTGAGAACCAAGACCC-3′ REV: 5′-AAGATGTCAAACTCACTCATGGC-3′ (141 bp); β-actin FOR: 5′-GCACTCTTCCAGCCTTCCTTCC-3′ REV: 5′-GAGCCGCCGATCCACACG-3′ (254 bp). All reactions were performed for 40 cycles using the following temperature profiles: 98 °C for 30 s; 57 °C for 20 s; and 72 °C for 30 s. The mRNA levels were normalized with β-actin mRNA levels, and the relative expression was calculated using the Ct value. PCR specificity was determined by both melting curve analysis and gel electrophoresis.

### Statistical Analysis

Data were analyzed with GraphPad Prism program (GraphPad Software Inc., San Diego, CA, USA). All data are represented as the mean ± SD. Statistical analysis was performed as indicated in figure legends. A *p*-value of <0.05 was considered to be statistically significant.

## Supporting Information Available

NMR spectra of compounds **1**–**5** (PDF)

## Dedication

Dedicated to Dr. Jon Clardy of Harvard Medical School for his pioneering work on bioactive natural products.
