# Supplementary material for: Antioxidant Activity of Compounds Isolated from Elaeagnus umbellata Promotes Human Gingival Fibroblast Well-Being
Source: J Nat Prod. 2020 Feb 7;83(3):626–37. doi: 10.1021/acs.jnatprod.9b01030 (PMC7997630; doi:10.1021/acs.jnatprod.9b01030)

Supporting Information

Antioxidant Activity of Compounds Isolated from *Elaeagnus umbellata* Promotes Human Gingival Fibroblast Well-Being

Anna Maria Iannuzzi,[†] Chiara Giacomelli,[†,‡] Marinella De Leo,[†,‡] Deborah Pietrobono,[†] Fabiano Camangi,[§] Nunziatina De Tommasi,[*,⊥] Claudia Martini,[†,‡] Maria Letizia Trincavelli,[†,‡] and Alessandra Braca[†,‡]

[†]Dipartimento di Farmacia, Università di Pisa, via Bonanno 33, 56126 Pisa, Italy

[‡]Centro Interdipartimentale di Ricerca "Nutraceutica e Alimentazione per la Salute", Università di Pisa, via del Borghetto 80, 56124 Pisa, Italy

[§]Scuola Superiore Sant'Anna di Studi Universitari e di Perfezionamento, Piazza Martiri della Libertà 33, 56127 Pisa, Italy

[⊥]Dipartimento di Farmacia, Università degli Studi di Salerno, via Giovanni Paolo II 132, 84084 Fisciano (SA), Italy

Table of Contents

Figure S1. ^1H NMR spectrum of compound **1** (CD_3OD, 600 MHz)

Figure S2. COSY spectrum of compound **1** (CD_3OD, 600 MHz)

Figure S3. HSQC spectrum of compound **1** (CD_3OD, 600 MHz)

Figure S4. HMBC spectrum of compound **1** (CD_3OD, 600 MHz)

Figure S5. ^1H NMR spectrum of compound **2** (CD_3OD, 600 MHz)

Figure S6. COSY spectrum of compound **2** (CD_3OD, 600 MHz)

Figure S7. HSQC spectrum of compound **2** (CD_3OD, 600 MHz)

Figure S8. HMBC spectrum of compound **2** (CD_3OD, 600 MHz)

Figure S9. ^1H NMR spectrum of compound **3** (CD_3OD, 600 MHz)

Figure S10. COSY spectrum of compound **3** (CD_3OD, 600 MHz)

Figure S11. HSQC spectrum of compound **3** (CD_3OD, 600 MHz)

Figure S12. HMBC spectrum of compound **3** (CD_3OD, 600 MHz)

Figure S13. ^1H NMR spectrum of compound **4** (CD_3OD, 600 MHz)

Figure S14. COSY spectrum of compound **4** (CD_3OD, 600 MHz)

Figure S15. HSQC spectrum of compound **4** (CD_3OD, 600 MHz)

Figure S16. HMBC spectrum of compound **4** (CD_3OD, 600 MHz)

Figure S17. ^1H NMR spectrum of compound **5** (CD_3OD, 600 MHz)

Figure S18. COSY spectrum of compound **5** (CD_3OD, 600 MHz)

Figure S19. HSQC spectrum of compound **5** (CD_3OD, 600 MHz)

Figure S20. HMBC spectrum of compound **5** (CD_3OD, 600 MHz)

Figure S1.

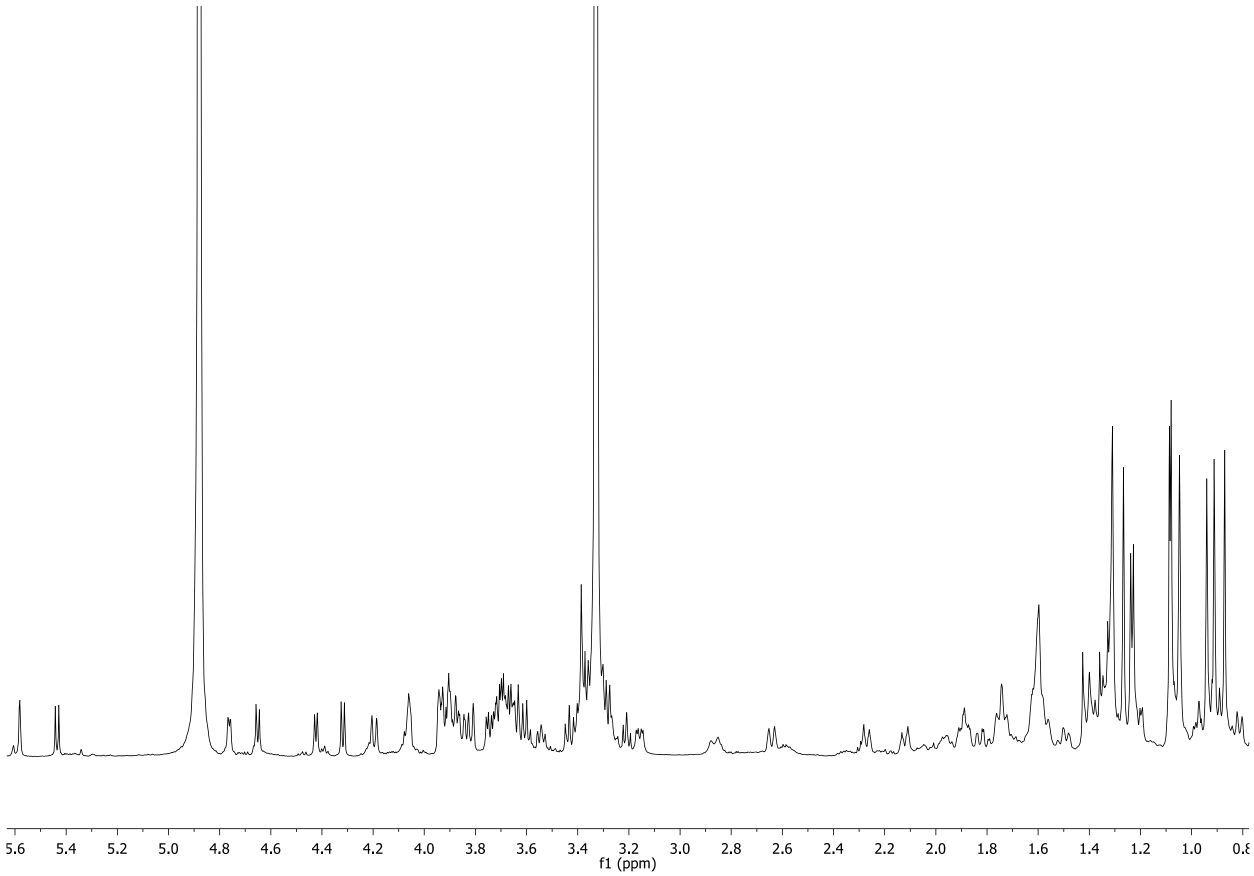

Figure S2.

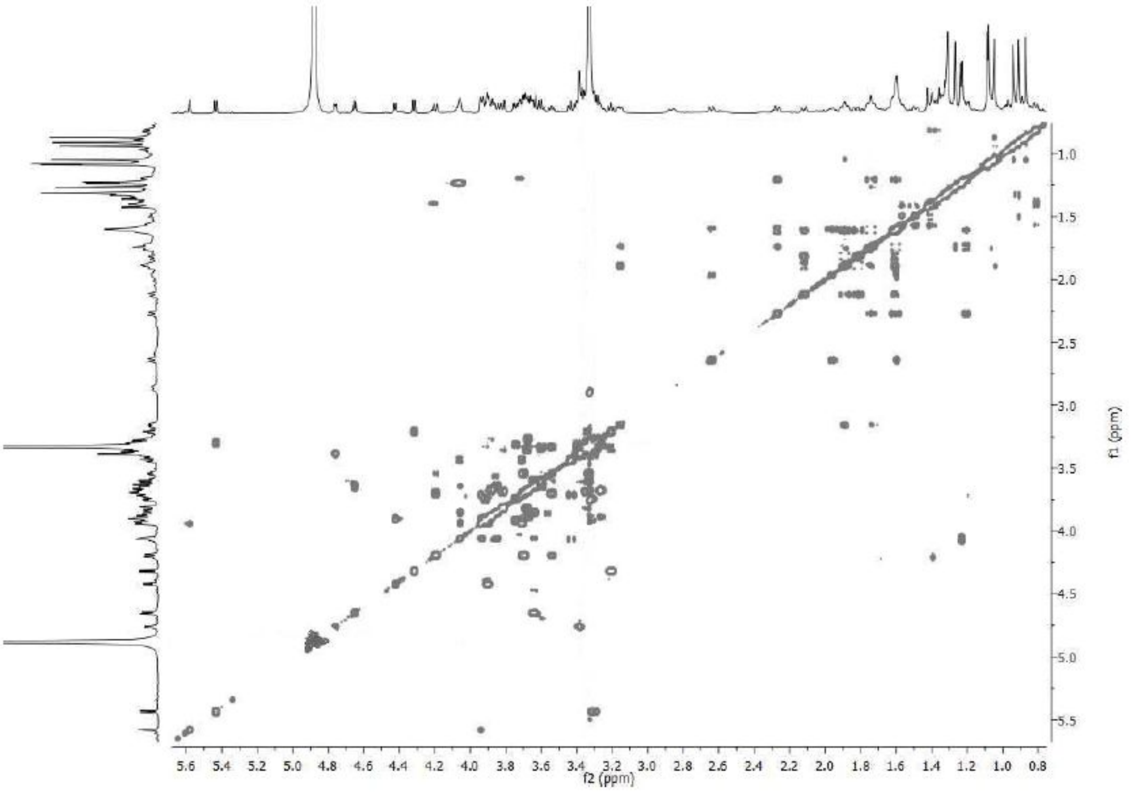

Figure S3.

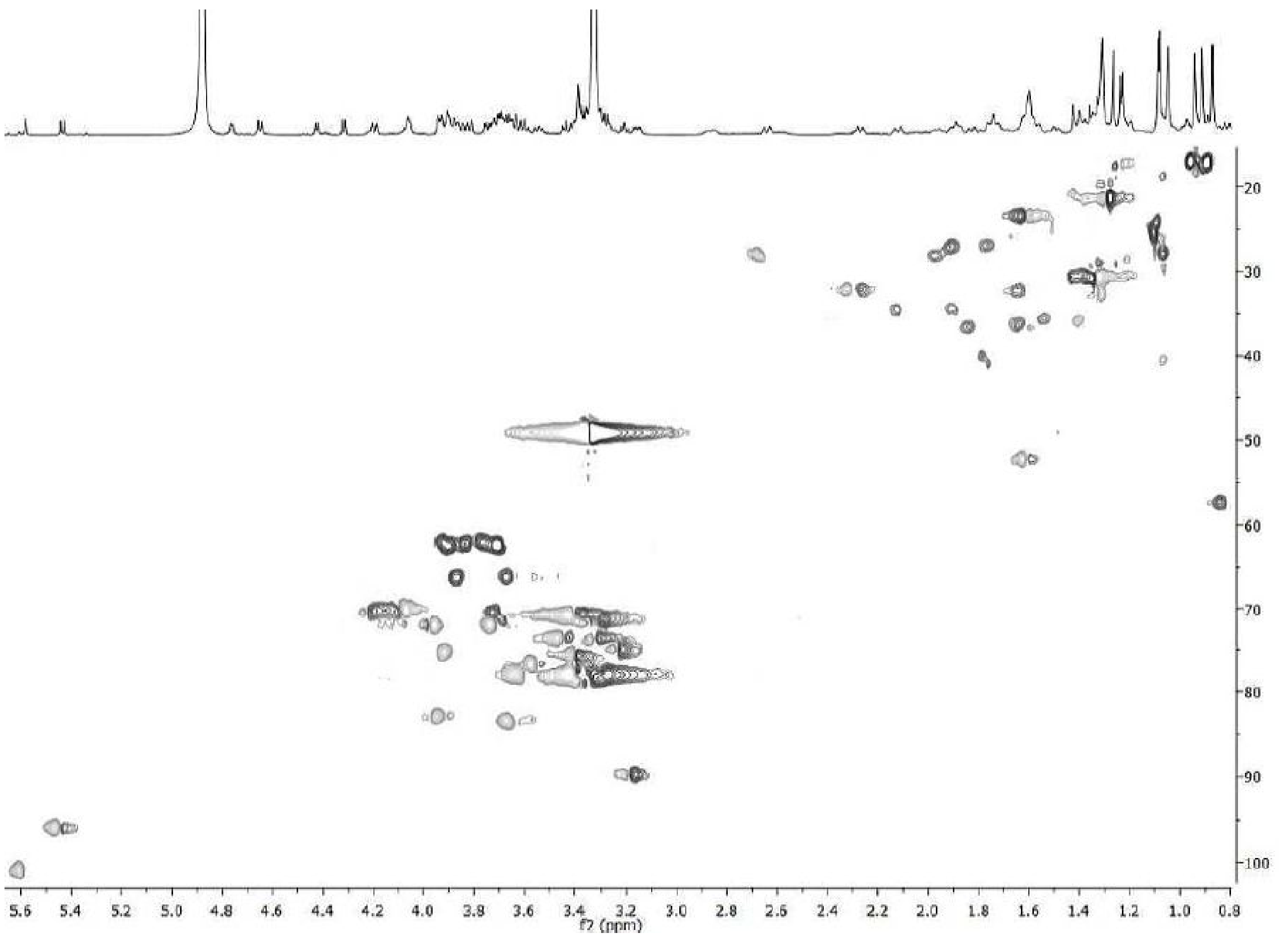

Figure S4.

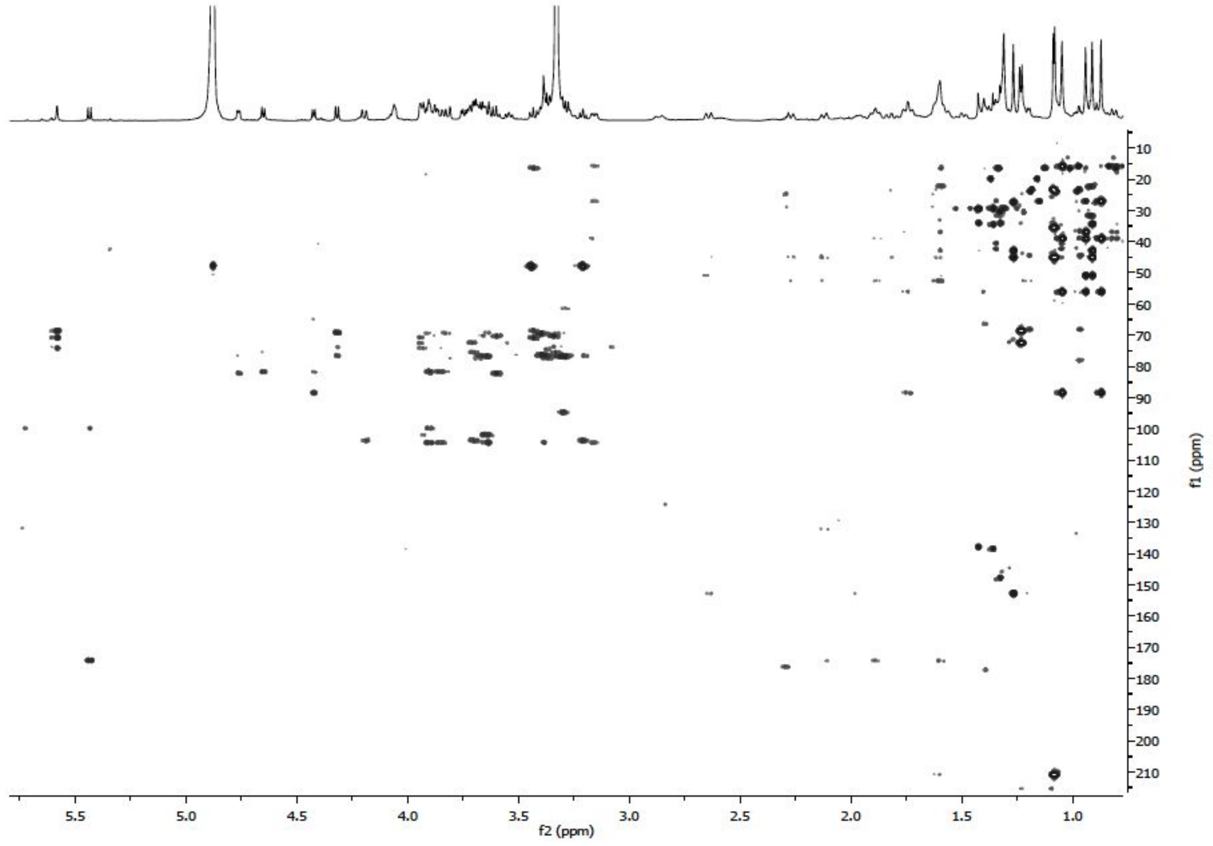

Figure S5.

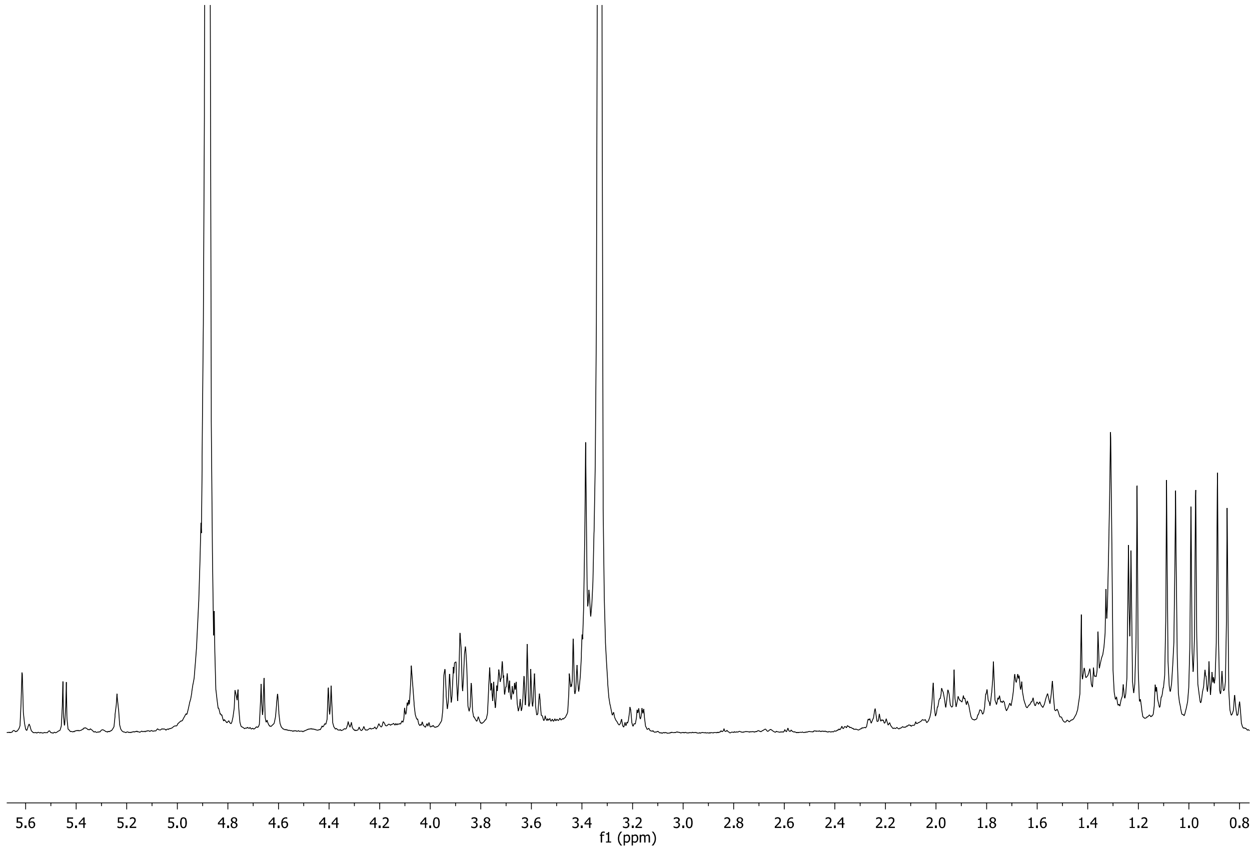

Figure S6.

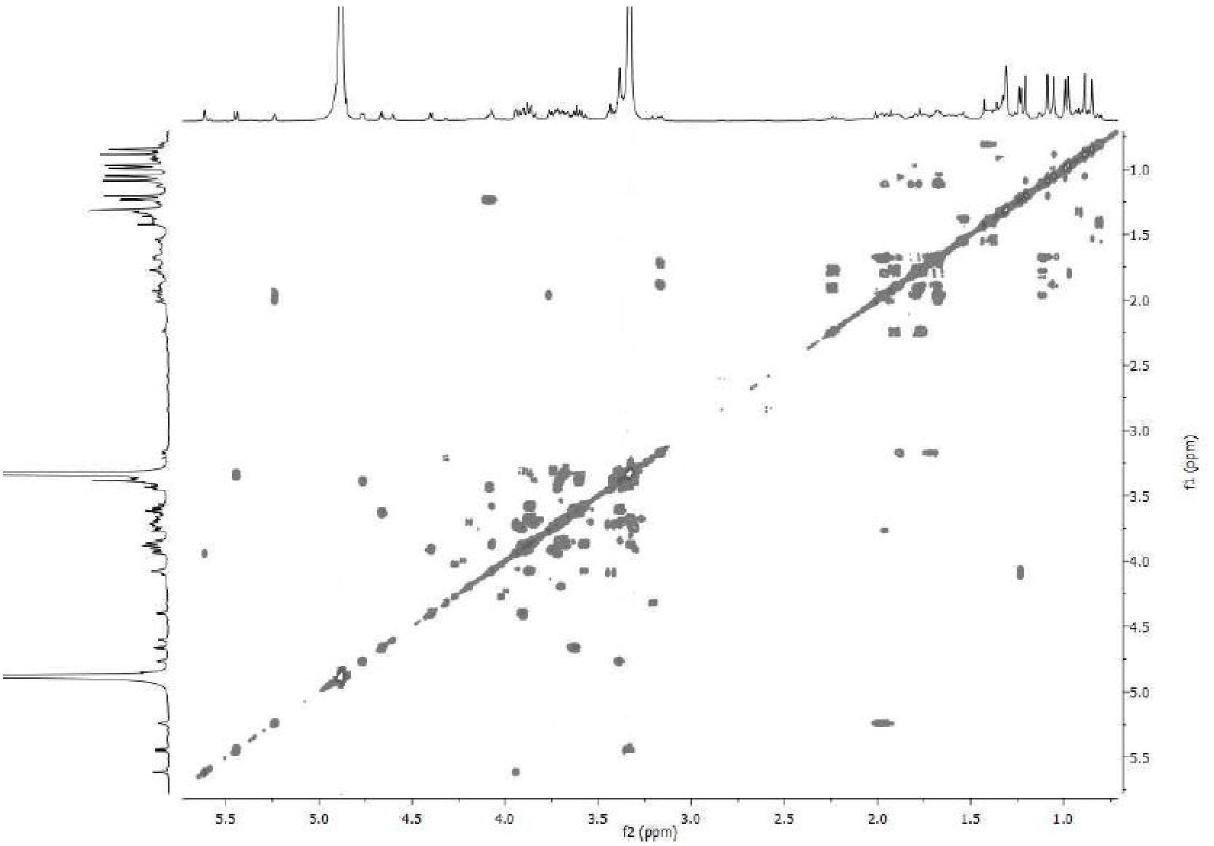

Figure S7.

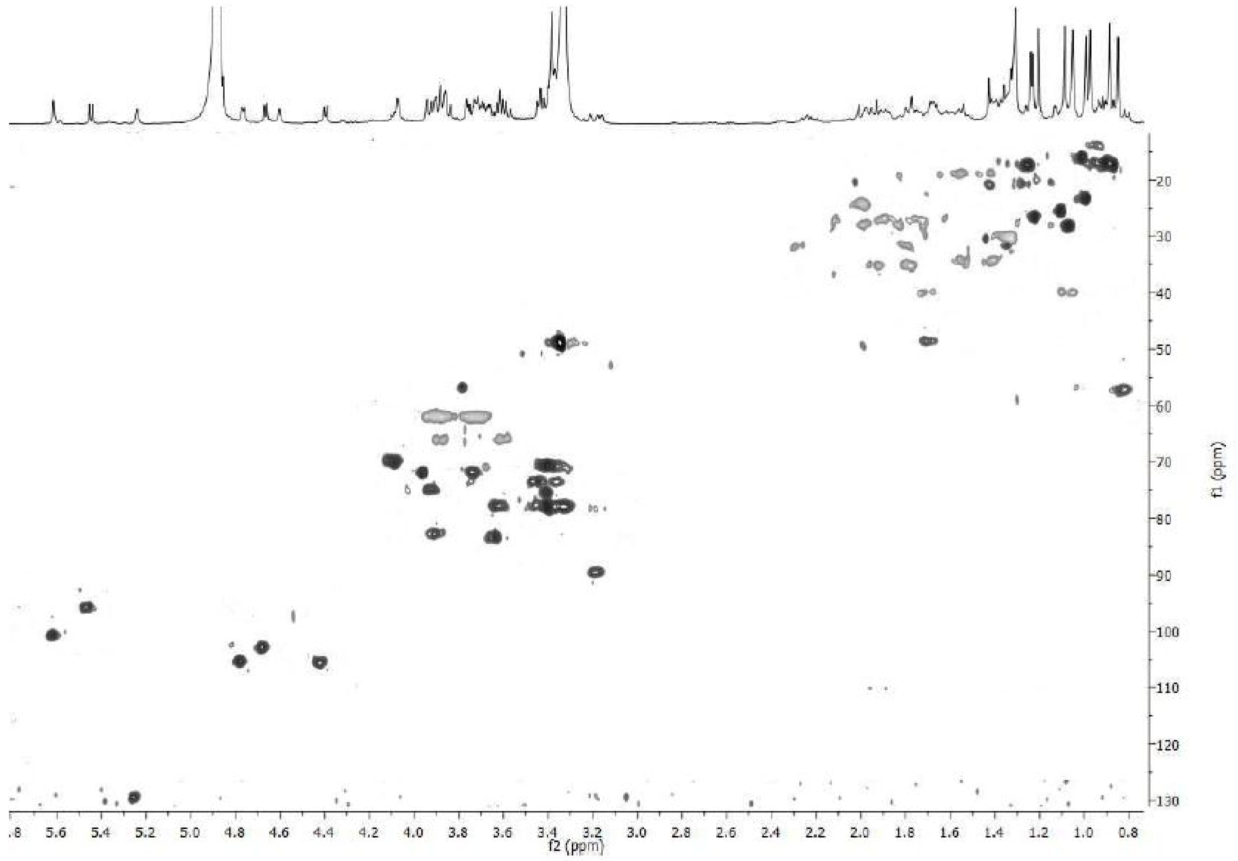

Figure S8.

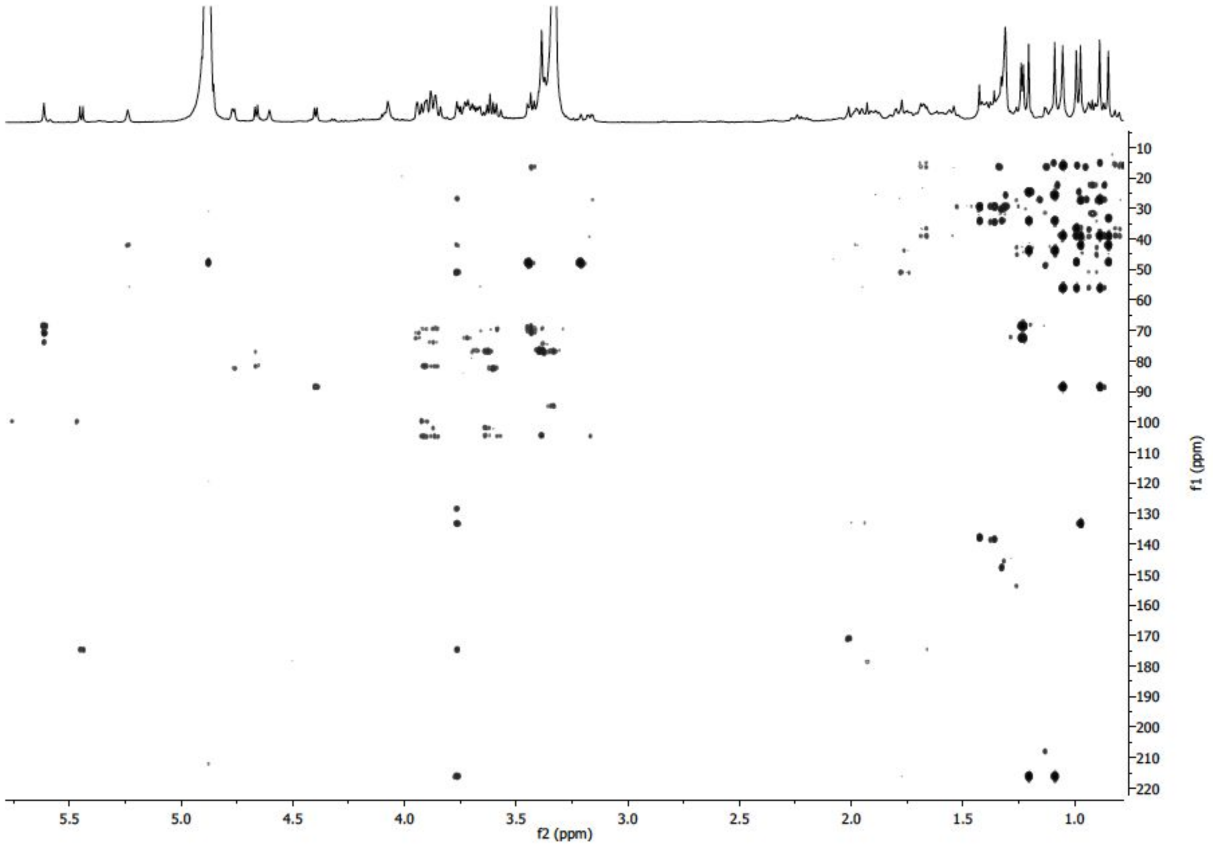

Figure S9.

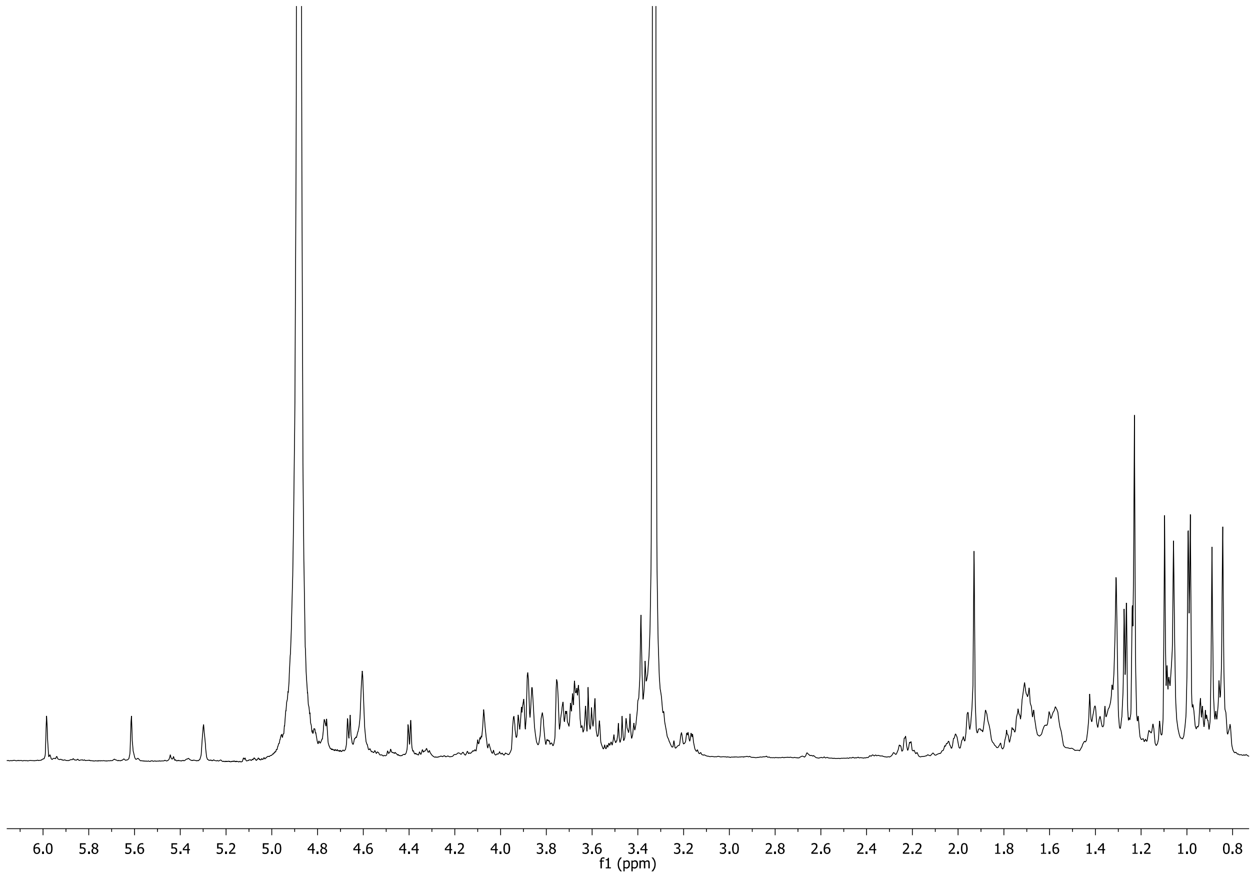

Figure S10.

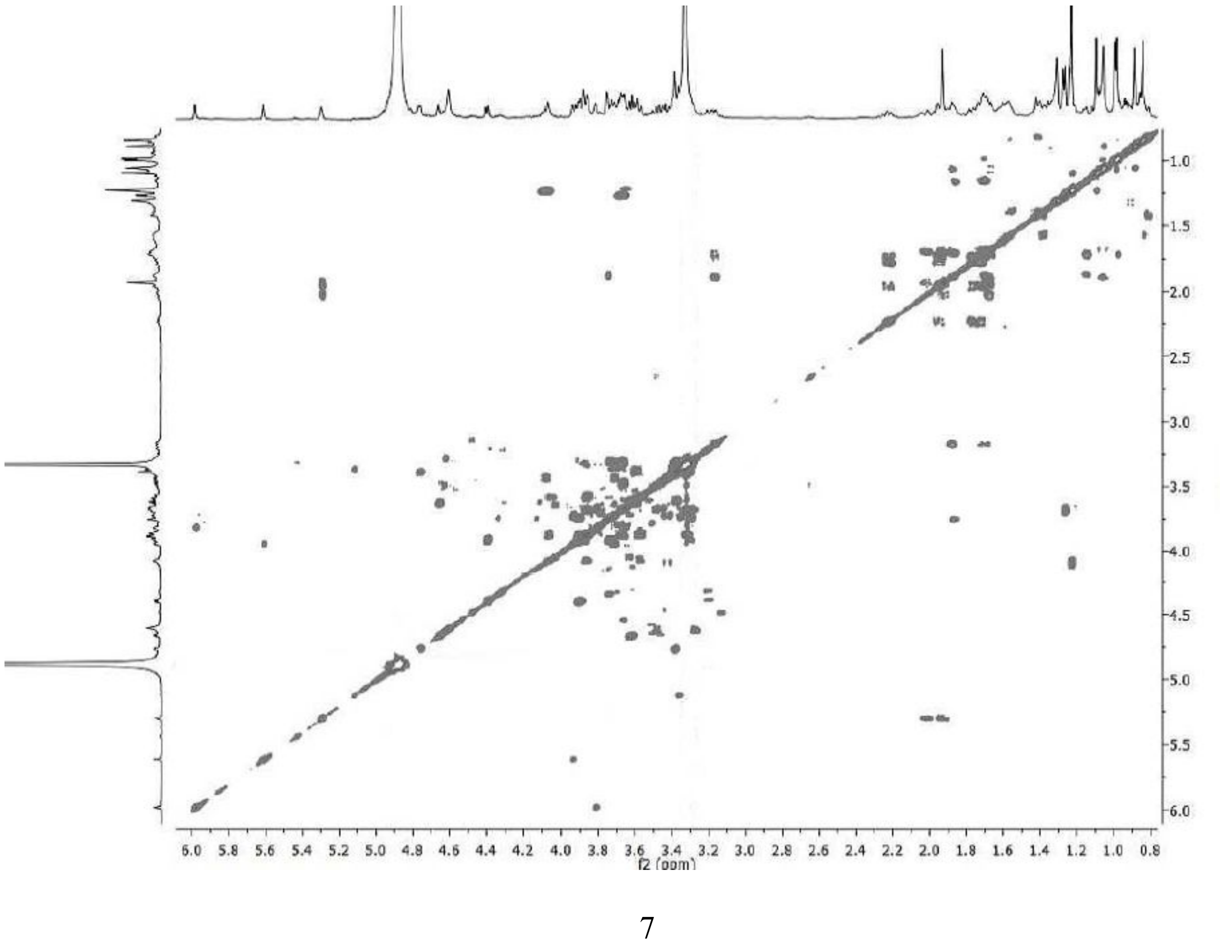

Figure S11.

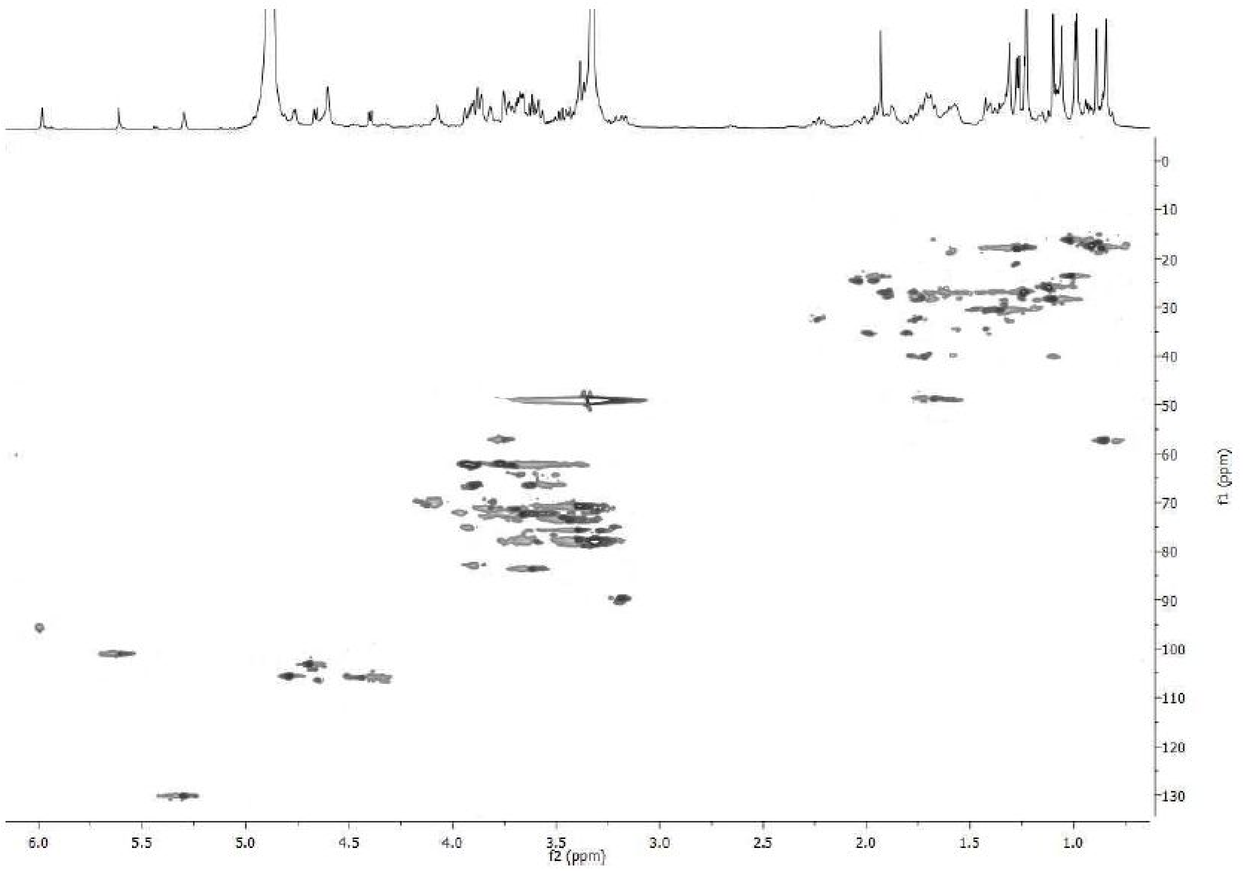

Figure S12.

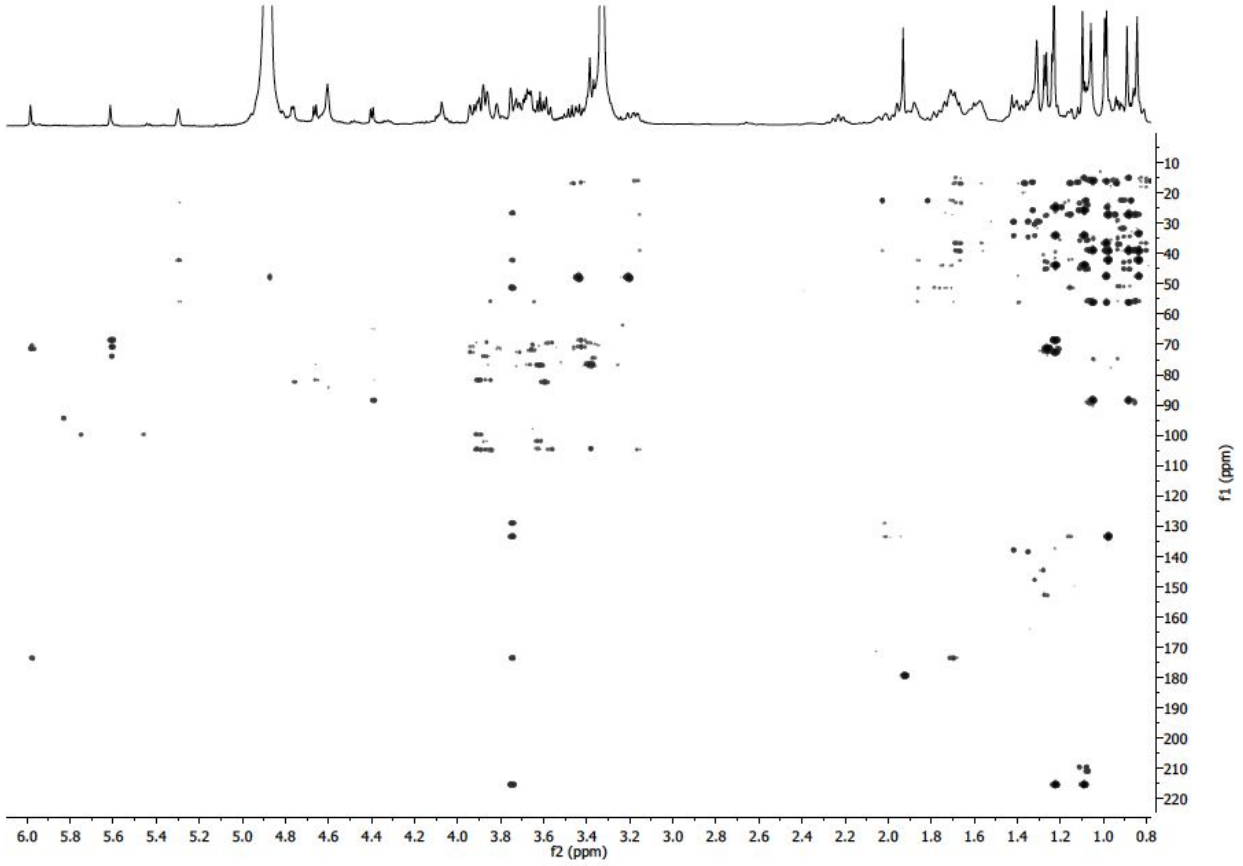

Figure S13.

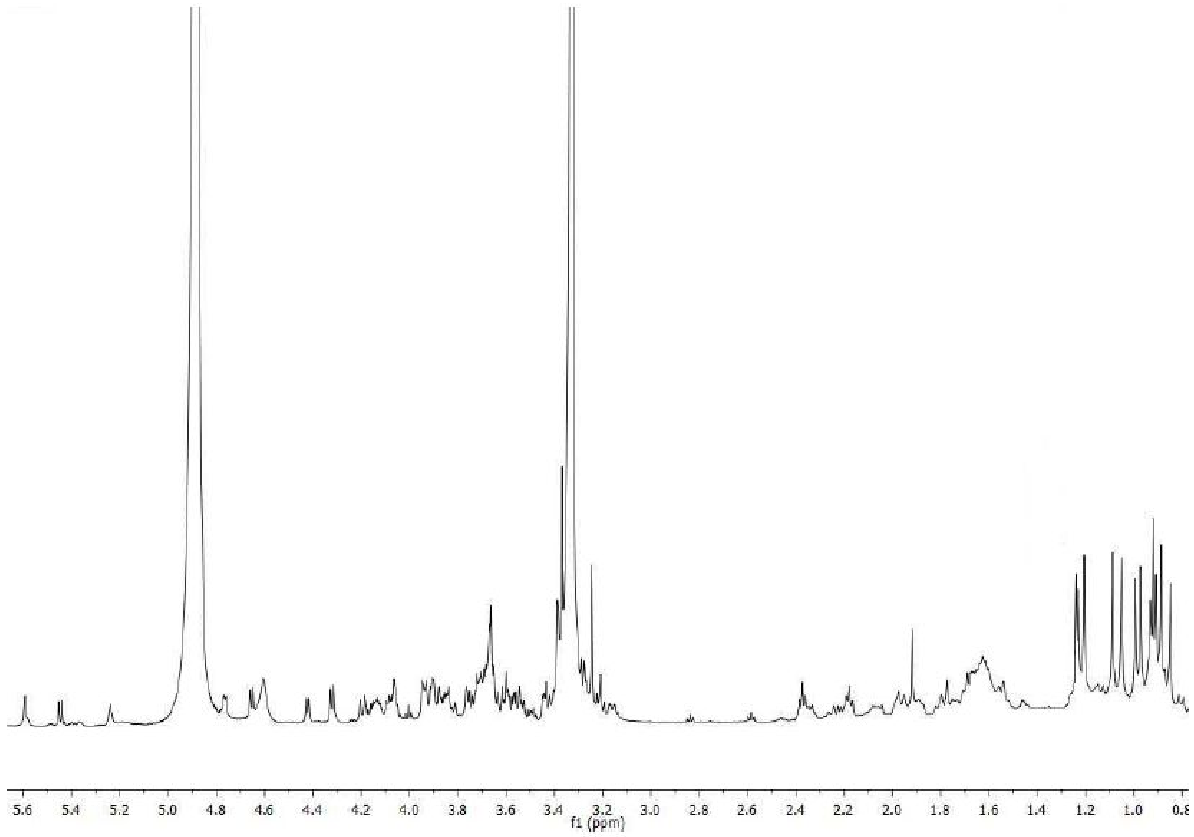

Figure S14.

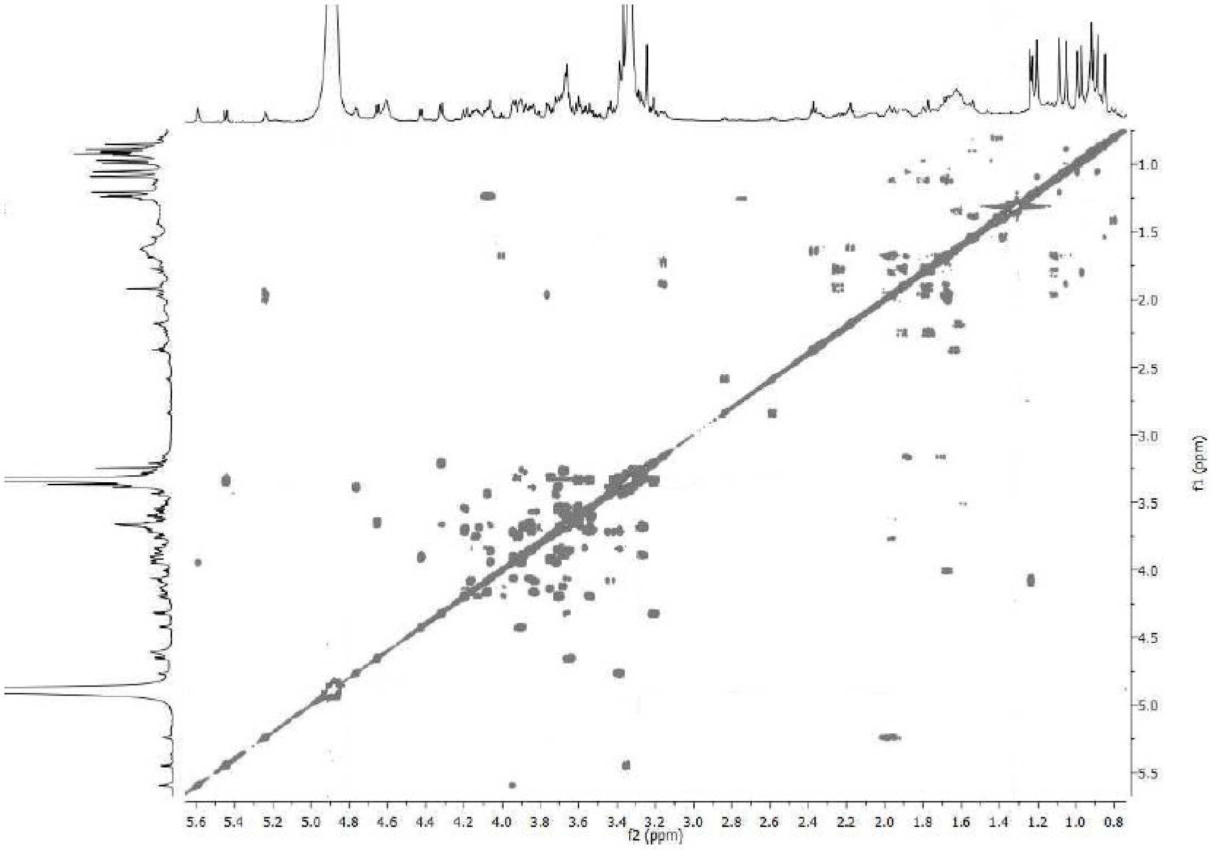

Figure S15.

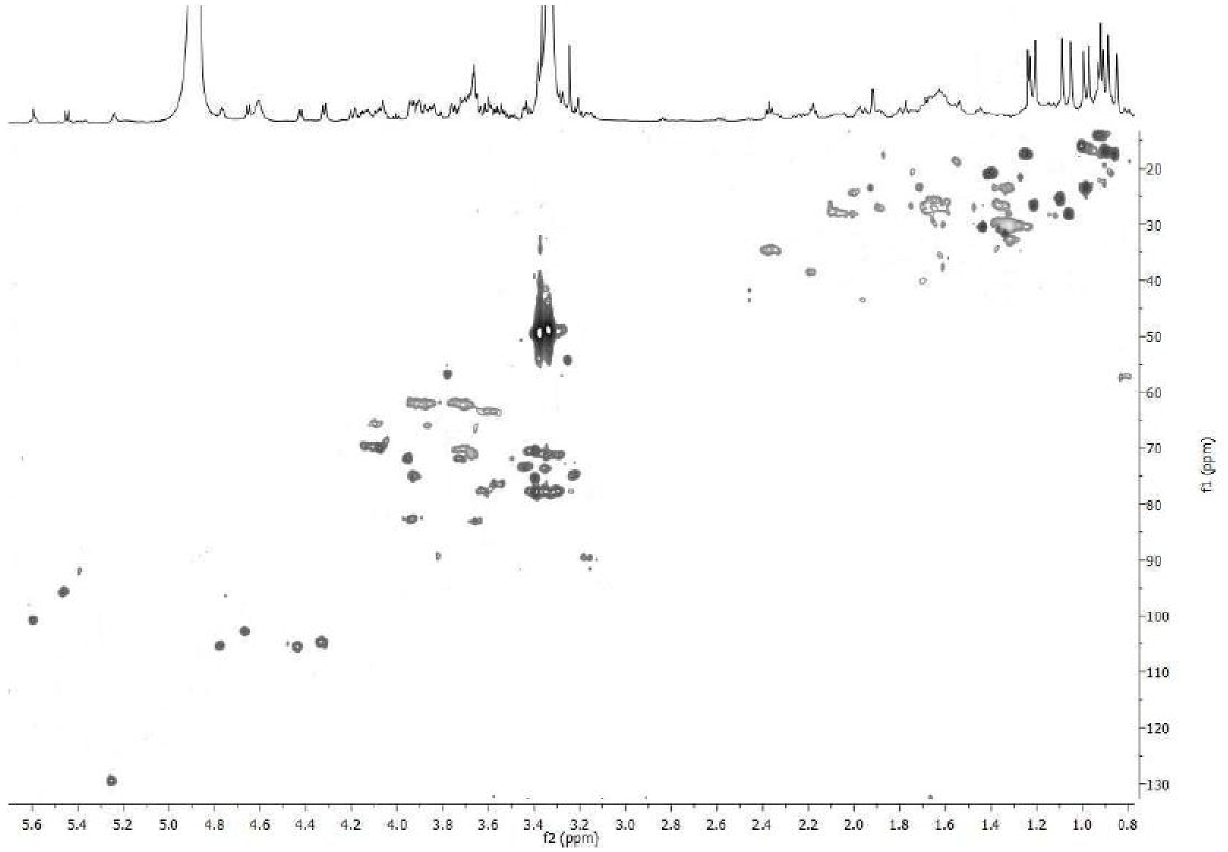

Figure S16.

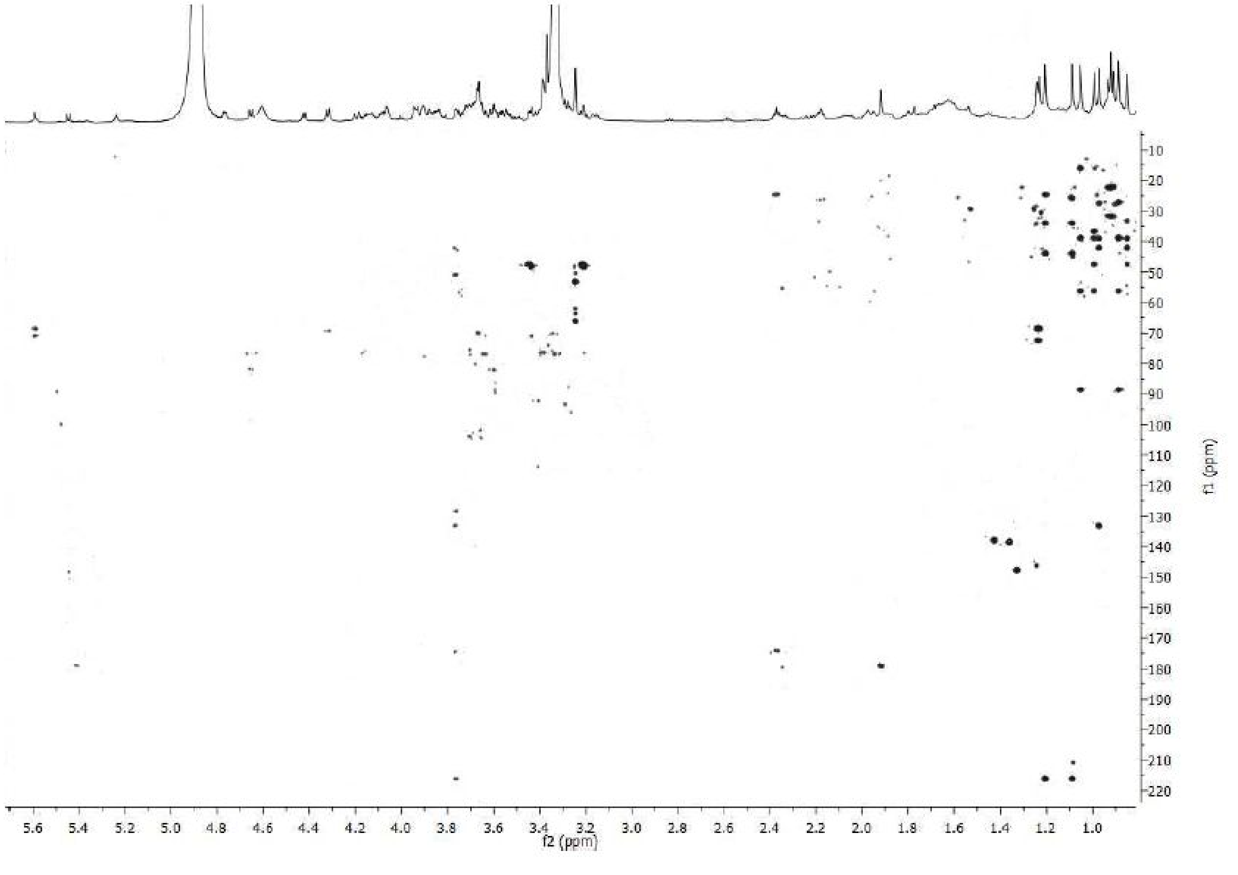

Figure S17

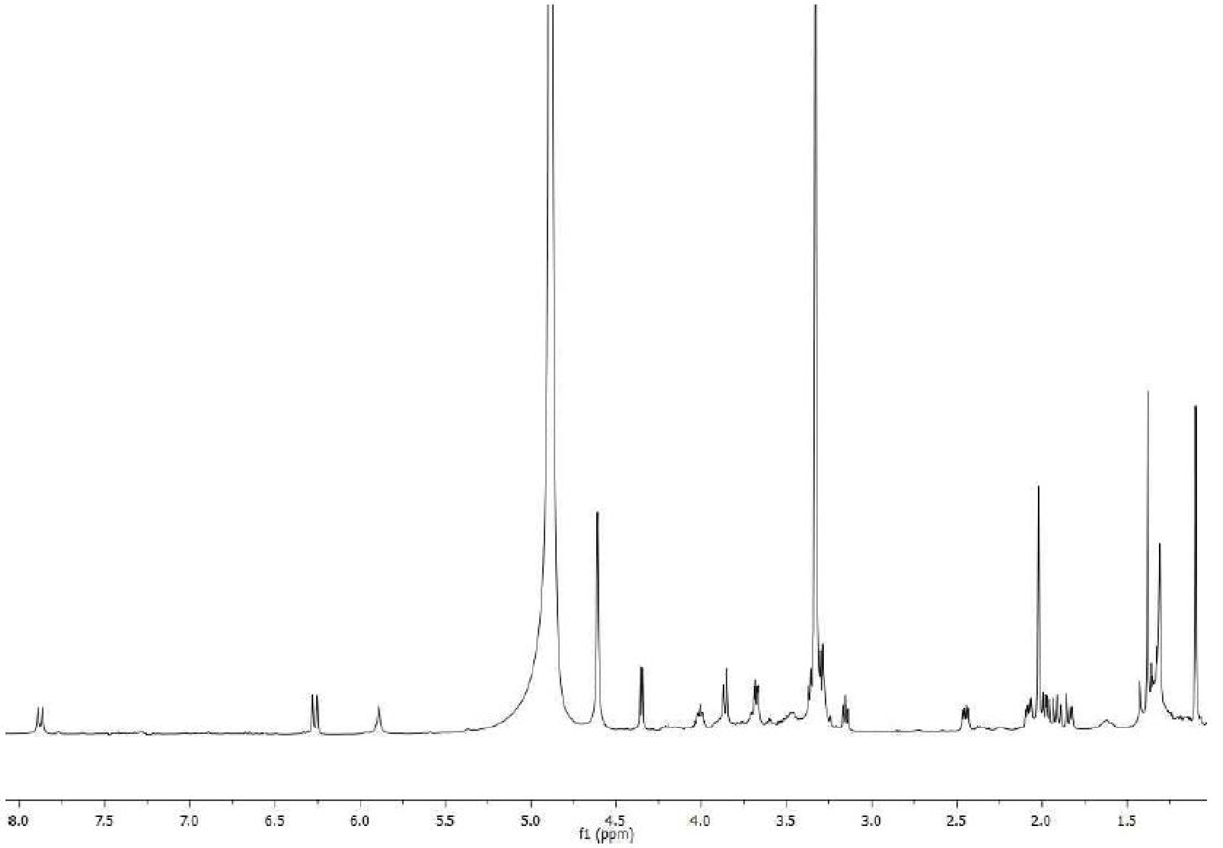

Figure S18

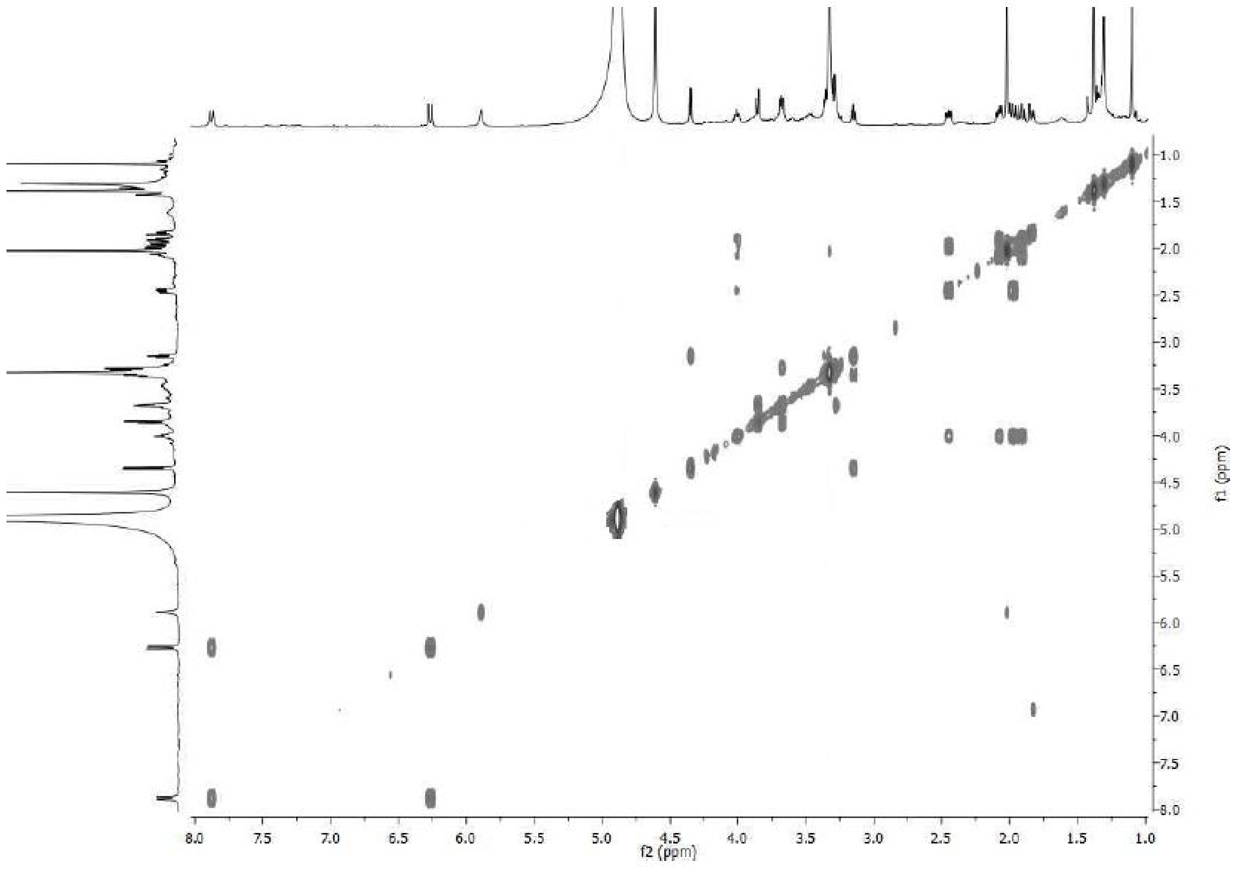

Figure S19

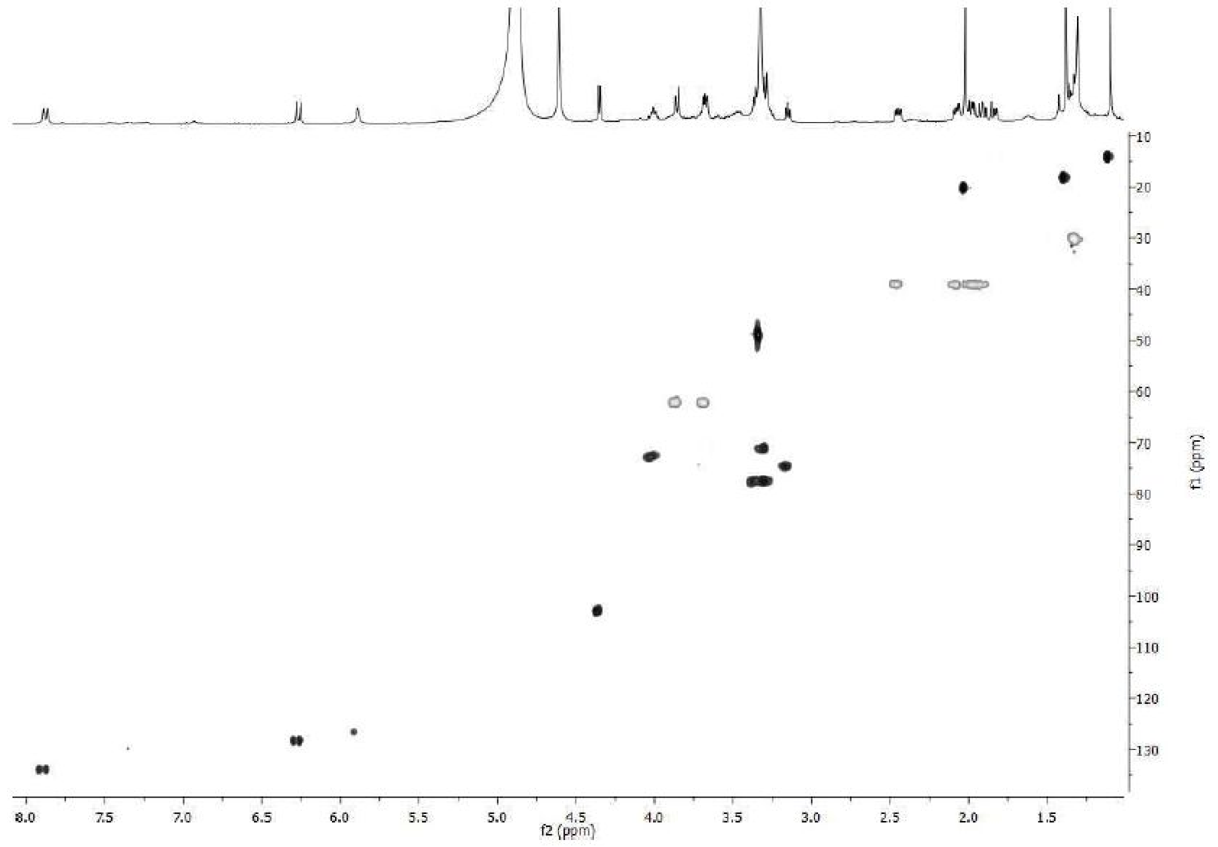

Figure S20

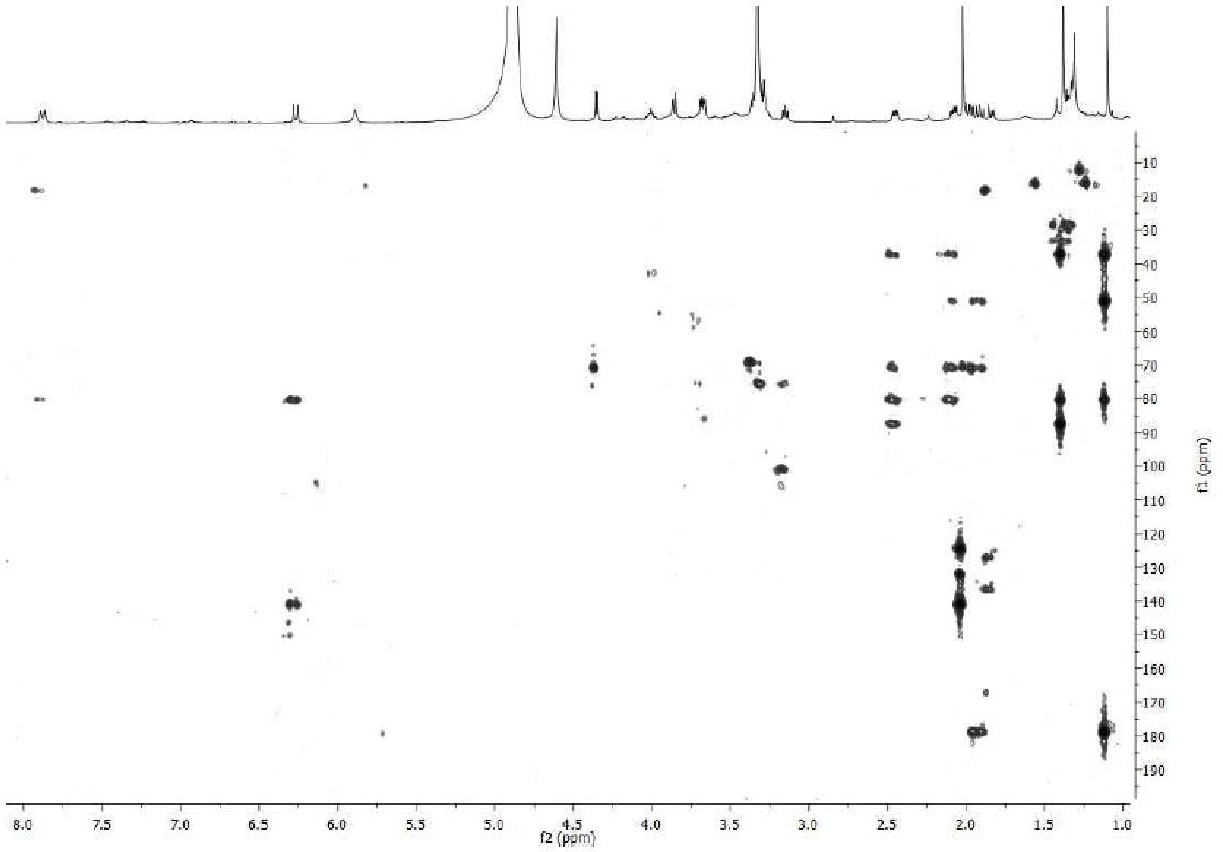

Supplement: Supplementary file 1 — np9b01030_si_001.pdf [file np9b01030_si_001.pdf]
